# Inhibitory activity of *Enhydra fluctuans* Lour. on calcium oxalate crystallisation through *in silico* and *in vitro* studies

**DOI:** 10.3389/fphar.2022.982419

**Published:** 2023-01-20

**Authors:** Bornika Chattaraj, Arijit Nandi, Anwesha Das, Amit Sharma, Yadu Nandan Dey, Dharmendra Kumar, Mogana R

**Affiliations:** ^1^ Department of Pharmacology, Dr. B.C. Roy College of Pharmacy and Allied Health Sciences, Durgapur, India; ^2^ Department of Pharmacy, Indira Gandhi National Tribal University, Lalpur, India; ^3^ Department of Pharmaceutical Sciences and Technology, Birla Institute of Technology, Mesra, Ranchi, India; ^4^ Narayan Institute of Pharmacy, Gopal Narayan Singh University, Sasaram, India; ^5^ Department of Pharmaceutical Biology, Faculty of Pharmaceutical Sciences, UCSI Education SDN.BHD, UCSI University, Kuala Lumpur, Malaysia

**Keywords:** calcium oxalate, crystallization, *Enhydra fluctuans*, Asteraceae, molecular docking, kidney stones

## Abstract

The decoction of the whole plant of *Enhydra fluctuans* is used ethno medicinally by various tribes for the treatment of kidney stones and urinary problems. However, no scientific studies were carried out to delineate its influence on urinary stone formation and crystallisation. Hence, the present study is proposed to investigate the effect of the aqueous extract of *Enhydra fluctuans* extract on *in vitro* crystallisation of calcium oxalate. The present study also evaluated. *in silico* studies of the metabolites with the target proteins present in the renal calcium oxalate stone matrix. The plant material was subjected to decoction to obtain an aqueous extract. The effect of the extract on calcium oxalate crystallization was evaluated by *in vitro* nucleation and aggregation assays. Further, the metabolites present in *E. fluctuans* were mined from the existing literature and their number was found to be 35. The selected 35 metabolites of *E. fluctuans* were subjected to molecular docking with the 5 proteins which are known to be responsible for calcium oxalate crystal growth. Results of *in vitro* studies indicated that the extract (50, 100, and 200 μg/mL) and standard drug cystone (1,000 μg/mL) exhibited an inhibitory role in the nucleation process where the percentage inhibitions were 52.69, 43.47, 21.98, and 31.67 μg/mL respectively. The results of molecular docking studies revealed that 2 out of 35 metabolites i.e. Baicalein-7-O-diglucoside and 4′,5,6,7-Tetrahydroxy-8-methoxy isoflavone-7-O-beta-D- galactopyranosyl-(1→3)-O-beta-D-xylopyranosyl-(1→4)- O-alpha-L-rhamnopyranoside showed modulatory effects on the four renal stone matrix-associated protein (Human CTP: Phosphoethanolamine Cytidylyltransferase (Protein Data Bank ID: 3ELB), UDP glucose: glycoprotein glucosyltransferase 2 (Gene: UGGT2) (AlphaFold) and RIMS-binding protein 3A (Gene: RIMBP3) (AlphaFold), and Ras GTPase activating-like protein (PDB: 3FAY) based on their docking scores which indicates that they may inhibit the crystallization process. Findings from this study show that *Enhydra fluctuans* may be effective in the prevention of the crystallization of calcium oxalate. However, further, *in vivo* studies as well as molecular studies are needed to be conducted to confirm and strengthen its anti-urolithiatic activity and to elucidate the possible mechanism of action involved therein.

## 1 Introduction

Urolithiasis is the accumulation of stones in the urinary system. The epidemiological report shows that 10%–12% of the population of industrial regions is getting affected due to urolithiasis (Ghodasara et al., 2010). Among all the kidney stones that occur in humans, calcium oxalate (CaOx) stones are more common and constitute about 80% of cases (Parmar, 2004). The current therapeutic antilithiatic agents include thiazide or alkali-citrate but their uses are limited due to their multiple adverse effects e.g., hypokalemia, hyponatremia, metabolic alkalosis, hypercalcemia, hyperglycemia, hyperuricemia, hyperlipidemia, and sulfonamide allergy (Pak, 1989). Additionally, the research on traditional medicines especially in kidney stones is rising tremendously in the last decade due to the scarcity of modern pharmacotherapy. Many medicinal plants were reported to be useful in the treatment of urolithiasis by various experimental studies (Sharma et al., 2016; Sikarwar et al., 2017). Though; traditional and folkloric medicines are highly efficacious, their uses are limited due to a lack of knowledge and scientific evidence for their probable molecular mechanism due to the presence of multi constituents in them (Atanasov et al., 2021; Dey et al., 2016). Previous literature suggests that the plants belonging to the Asteraceae family are known to have antilithiatic properties and are being used for years by many tribal people all over the world in their ethnomedicinal practices. (Ahmed et al., 2017).

*Enhydra fluctuans* Lour. (Synonym: Helencha) belongs to the family Asteraceae, is a well-known plant which is mainly found in India, Bangladesh, Srilanka, Burma, and other south-east Asian countries. In ethnomedicinal practices, the decoction of the whole plant of *E. fluctuans* is consumed by various tribes of the Northeast region of India and Bangladesh for the treatment of kidney stones and urinary problems (Lokendrajit et al., 2011; Ahmed et al., 2016; Ahmed et al., 2017). However, no scientific studies were carried out to delineate its influence on experimentally-induced urolithiasis. Physiologically, crystallization of the stones is the first step in the etiology of urolithiasis which begins with increased urinary supersaturation and subsequent formation of the solid crystalline particles within the urinary tract. These crystals then grow and aggregate with other crystals in solution, and are ultimately retained and accumulated in the kidney (Basavaraj et al., 2007; Kok et al., 1990). Therefore, if this progression of crystallization can be prevented, then urolithiasis can also be prevented. Further, CaOx crystals are supported in the renal stone matrix in the kidney where various types of proteins are present which play a major role in the adhesion of the kidney stone crystals and also modulate the crystalline process (Aggarwal et al., 2013; Boyce and Garvey. 1956; Khanand Kok. 2004). Though these proteins play a potential role in crystal-membrane interaction, crystal growth, and stone formation but their mechanism in urolithiasis is unexplored. Five such proteins which were isolated from human renal CaOx were identified as Ethanolamine-phosphate cytidylyltransferase, Ras GTPase-activating-like protein, UDP-glucose: glycoprotein glucosyltransferase 2, RIMS-binding protein 3A and Macrophage-capping protein (Aggarwal et al., 2013). Hence, the present study investigated the effect of an aqueous extract of *E. fluctuans* (AEEF) on the *in-vitro* crystallization of CaOx and *in silico* studies through the molecular docking analysis of the metabolites with the human renal CaOx stone matrix proteins involved in the CaOx stone formation. All the studies were conducted by following the guidelines mentioning the four pillars of best practice in ethnopharmacology (Heinrich et al., 2020; Heinrich et al., 2022).

## 2 Materials and methods

### 2.1 Drugs and chemicals

Calcium chloride dihydrate (CaCl_2_H_4_O_2_), Sodium oxalate (Na_2_C_2_O_4_), Sodium chloride (NaCl), and Sodium acetate trihydrate (CH_3_COONa·3H_2_O) were procured from HiMedia Laboratories Private Limited while Cystone was procured from Himalaya Wellness Company. All the other chemicals which were in this study were of the highest purity grade.

### 2.2 Collection and processing of the plant material

The plant material was collected in the month of November 2021 from the local market of Durgapur, West Bengal, India. The plant material was identified by Dr. R. K. Gupta, Taxonomist and Scientist-E, Central National Herbarium, Botanical Survey of India (BSI) and it has been identified as *Enhydra fluctuans* Lour belongs to the family Asteraceae. A voucher specimen (BC-01) of the authenticated plant has been deposited on dated 12/01/2022 in the herbarium of BSI.

### 2.3 Extraction of *Enhydra fluctuans*

Fresh plant materials of *E. fluctuans* were washed to eliminate soil and other contaminants. They were cut into small pieces. 20 g from it was taken into a beaker containing 400 mL of distilled water and subjected to decoction for 4 h at 100°C. After 4 h, the extract was filtered through Whatman filter paper no.1 and concentrated under vacuum to obtain aqueous extract (AEEF) and stored in a vacuum desiccator. The aqueous extract of dark green color and dry sticky consistency was obtained with a percentage yield of 18%.

### 2.4 *In vitro* crystallization of calcium oxalate: Nucleation and aggregation assay

Nucleation and aggregation assay was carried out by using the previously described method (Hess et al., 2000) with slight modification. Briefly, the solutions of 10 mM calcium chloride dihydrate and 1 mM sodium oxalate, containing 200 mM NaCl and 10 mM sodium acetate trihydrate, were freshly prepared and the pH was adjusted to 5.7. The temperature was maintained at 37°C in the water bath. 25 mL of sodium oxalate solution was placed in a beaker and was kept in a hot plate at 37°C with a magnetic stirrer (800 rpm) (REMI Sales and Engineering Ltd.). Then, 1 mL of distilled water/standard cystone (1,000 μg/mL)/AEEF (50, 100, and 200 μg/mL) was added and lastly, 25 mL of calcium chloride solution was added in the solution. The absorbance of the resultant solutions was measured at 620 nm in a UV Visible spectrophotometer (Shimadzu UV-1800), on every 15 s over 30 min. The whole experiment was performed in triplicate and then the final solution was observed under a light microscope. The percentage inhibition of CaOx crystallization in the presence of cystone or AEEF was calculated by the formula mentioned below.

Percentage inhibition = [1-(Tsi/Tsc)] × 100.

Here, Tsc is the turbidity slope of the control and Tsi is the turbidity slope in the presence of the cystone/AEEF.

### 2.5 In silico studies

#### 2.5.1 Binding pocket analysis

Schrödinger LLC’s SiteMap module was utilized to analyze and predict the binding site present in the protein (Halgren, 2009). With the help of this tool, the binding pockets of any protein can be investigated and then predicted by using grid points, which are known as site points, followed by employing a probe’s electrostatic and Van der Waals (VdW) interactions located at every point for creating field maps. This probe also performs 1.6 Å-VdW radius-containing water molecule simulation. In principle, SiteMap separates the protein surface that is accessible to the solvent into three classes of regions, namely hydrophilic, hydrophobic, and balanced-characterized regions, among which the hydrophilic region is further classified into H-bond acceptor, donor, and metal-binding regions. The regions containing H-bond acceptors and donors refer to a ligand’s H-bond accepting and donating degree, respectively.

#### 2.5.2 Molecular docking

The metabolites present in *E. fluctuans* were mined from the existing literature and their number was found to be 35. The selected 35 metabolites of *E. fluctuans* were subjected to molecular docking with the 5 proteins which are known to be responsible for calcium oxalate crystal growth. Aggarwal et al. (2013) isolated five proteins from the human renal CaOx stone matrix which are responsible for the crystal-membrane interaction, crystal growth, and stone formation which are Ethanolamine-phosphate cytidylyltransferase, Ras GTPase-activating-like protein, UDP-glucose: glycoprotein glucosyltransferase 2, RIMS-binding protein 3A and Macrophage-capping protein. Hence, the selected 35 metabolites of *E. fluctuans* were subjected to molecular docking with the 5 proteins. The crystal structure of Ethanolamine-phosphate cytidylyltransferase (PDB ID: 3ELB) (Aggarwal et al., 2013), Macrophage-capping protein (PDB ID: 1J72) (Vorobiev et al., 2011), and Ras GTPase-activating-like protein (PDB ID: 3FAY) (Kurella et al., 2009), were extracted from the RCSB Protein Data Bank (PDB). The PDB file of UDP glucose: Glycoprotein glucosyltransferase 2 (Gene: UGGT2) (AlphaFold) and RIMS-binding protein 3A (Gene: RIMBP3) (AlphaFold) were obtained from AlphaFold Protein Structure Database, AlphaFold-DeepMind (Jumper et al., 2021; Varadi et al., 2022). All the protein structures (crystallized and predicted) were then prepared using the Protein Preparation Wizard module in Schrodinger 2017_2 (Sastry et al., 2013) to remove all the water molecules (in case of co-crystallized ligand-bound protein i.e. 3ELB, 1J72, 3FAY water molecules remained only beyond 5 Å from the ligand molecule), missing side chains, missing loops, and hydrogen atoms were added, protonation states and partial charges were assigned using the OPLS3 force field. After that, all the protein structures were restrained-minimized until the root-mean-square deviation (RMSD) of the non-H atoms converged to .3 Å. The structures of the 35 metabolites of *E. fluctuans* were retrieved from the PubChem database and prepared using the LigPrep module of Schrodinger 2017-2 suite to generate tautomers, the chiralities were retained and the ionization state at pH 7.0 ± 2.0 was determined using Epik (Shelley et al., 2007). Using the prepared and minimized receptor structures, the receptor grids were generated either around the co-crystallized ligand site of the crystal structure or around the sitemap-generated sites of the proteins. Then, the 35 metabolites were docked with the proteins using the Glide XP protocol (Friesner et al., 2006). The PDB ID: 3ELB was validated through the docking of the co-crystallized ligand. For the other four PDB ID 1J72, and 3FAY were validated through molecular dynamics studies. Two other AlphaFold predicted proteins RIMS-binding protein 3A, and UDP glucose: glycoprotein glucosyltransferase 2 were also validated through docking studies.

#### 2.5.3. Molecular mechanics generalized born surface area (MMGBSA) calculation

The binding free energy was calculated from the structural information of the MMGBSA method. These approaches employ the solvent accessibility method, molecular mechanics, and the generalized born model to evade the free energy simulations’ convolution (Genheden and Ryde, 2015). The energy difference between the bound and unbound complex of the protein-ligand complex was calculated with the help of MMGBSA. The binding free energies were computed by the equation as follows

$$MMGBSA∆G_{Bind}{=G}_{Complex}-\left(G_{Receptor}{+G}_{Ligand}\right)$$

Here the MMGBSA calculation was done in Prime software (Jacobson et al., 2004) by considering the VSGB solvation model with OPLS3 Force Field with minimized sampling method.

#### 2.5.4 Molecular dynamics

The docked complexes of the protein-ligand were simulated in the Desmond module of Schrodinger software. An orthorhombic box was built using a system builder panel. A simple point charge model was used as a water model for the simulation of the docked ligand-protein complex. The constant-temperature, constant-pressure ensemble (NPT) was used for the MD simulation at a temperature of 310 K and an atmospheric pressure of 1.013 bar for 50 ns. The output of the molecular dynamics studies was studied in detail using the Simulation Interactions Diagram Report of the Desmond software (Bowers et al., 2006; Auti et al., 2022).

#### 2.5.5 In silico physicochemical and ADME/T studies

Qikprop module of Schrödinger suite 2017-2 (QikProp, 2017_2) was used to determine the physicochemical and ADME/T properties of the best two molecules which helps to determine the physicochemical significant descriptors and pharmacokinetically important properties of the molecules. For the fast assessment of the physicochemical and ADME/T properties of the Baicalein-7-O-diglucoside, and 4′,5,6,7-Tetrahydroxy-8-methoxy isoflavone-7-O-beta-D- galactopyranosyl-(1→3)-O-beta-D-xylopyranosyl-(1→4)- O-alpha-L-rhamnopyranosideit were discussed in the results section.

#### 2.5.6 Pharmacophore modeling

The PHASE module of Schrödinger Release 2017_2 was used for generating common features of the best two molecules viz, 4′,5,6,7-Tetrahydroxy-8-methoxy isoflavone-7-O-beta-D-galactopyranosyl-(1→3)-O-beta-D-xylopyranosyl-(1→4)-O-alpha-L-rhamnopyranoside and Baicalein-7-O-diglucoside, obtained by performing XP docking and MD simulations by selecting multiple ligands from the “Create pharmacophore model using” section considering both the ligands active. PHASE generally provides pharmacophoric features such as H-bond acceptor (A), donor (D), positively (P), and negatively (N) ionic, hydrophobic (H), and aromatic ring (R). Here, the pharmacophore method employed was “Find best alignment and common features” without generating any conformers, as the ligands were not pre-aligned. For the development of the pharmacophore hypothesis, a minimum of three to a maximum of five features were selected. All the other parameters remained the default (Dixon et al., 2006).

### 2.6 Statistical analysis

All the data were analyzed by linear regression analysis, wherever necessary.

## 3 Results and discussion

The main loophole in the medical management of urolithiasis is a recurrence of stone formation. The recurrence of hypercalciuria and hyperoxaluria is generally undergone through the drug treatment of thiazide as diuretics and alkali citrate (Pak, 1989). Surgical endoscopic stone removal has modernized the treatment of urolithiasis but it can increase the occurrence of new stone formation and the shock wave can cause renal injury, decreased renal function, and increased recurrence of lithiasis (50%–80%) (Kalayan et al., 2009). That is why, the effect of the whole plant of *E. fluctuans*, which is being used traditionally in the treatment of urolithiasis, was studied by using *in vitro* models of urolithiasis. Further, CaOx crystals are supported in the renal stone matrix in the kidney where the various types of proteins play a major role in the adhesion of the kidney stone crystal and also modulate the crystalline process (Aggarwal et al., 2013). Though these proteins play a potential role in crystal-membrane interaction, crystal growth, and stone formation, their mechanism in urolithiasis is unexplored. Hence, *in silico* studies like molecular docking analysis of the metabolites were also carried out with the human renal CaOx stone matrix proteins involved in the CaOx stone formation.

### 3.1 Effect on *in vitro* CaOx crystallisation

**FIGURE 1:**
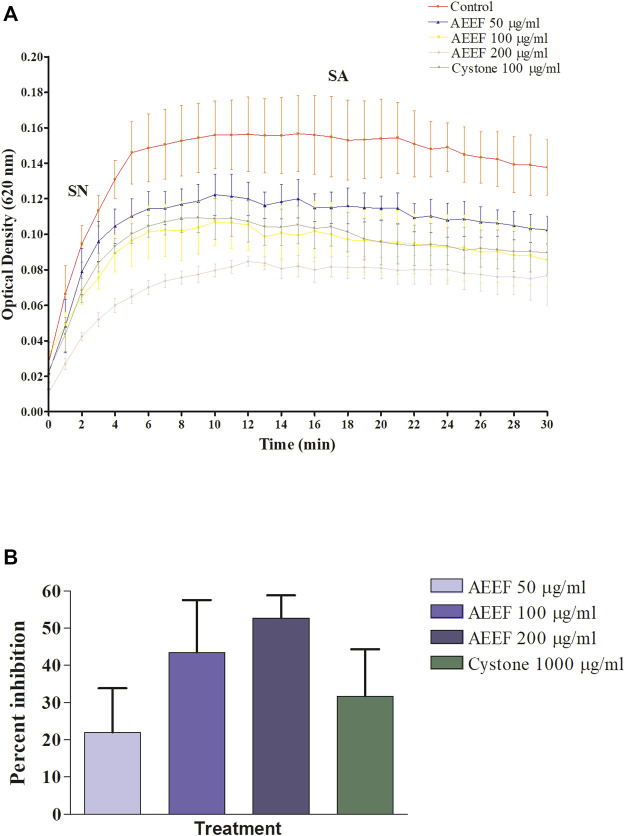
Effect of AEEF on CaOx crystallization **(A)** Change in the optical density of different solutions at different time intervals **(B)** Percent inhibition of nucleation. Values are expressed as mean ± S.D. (n = 3).

**FIGURE 2:**
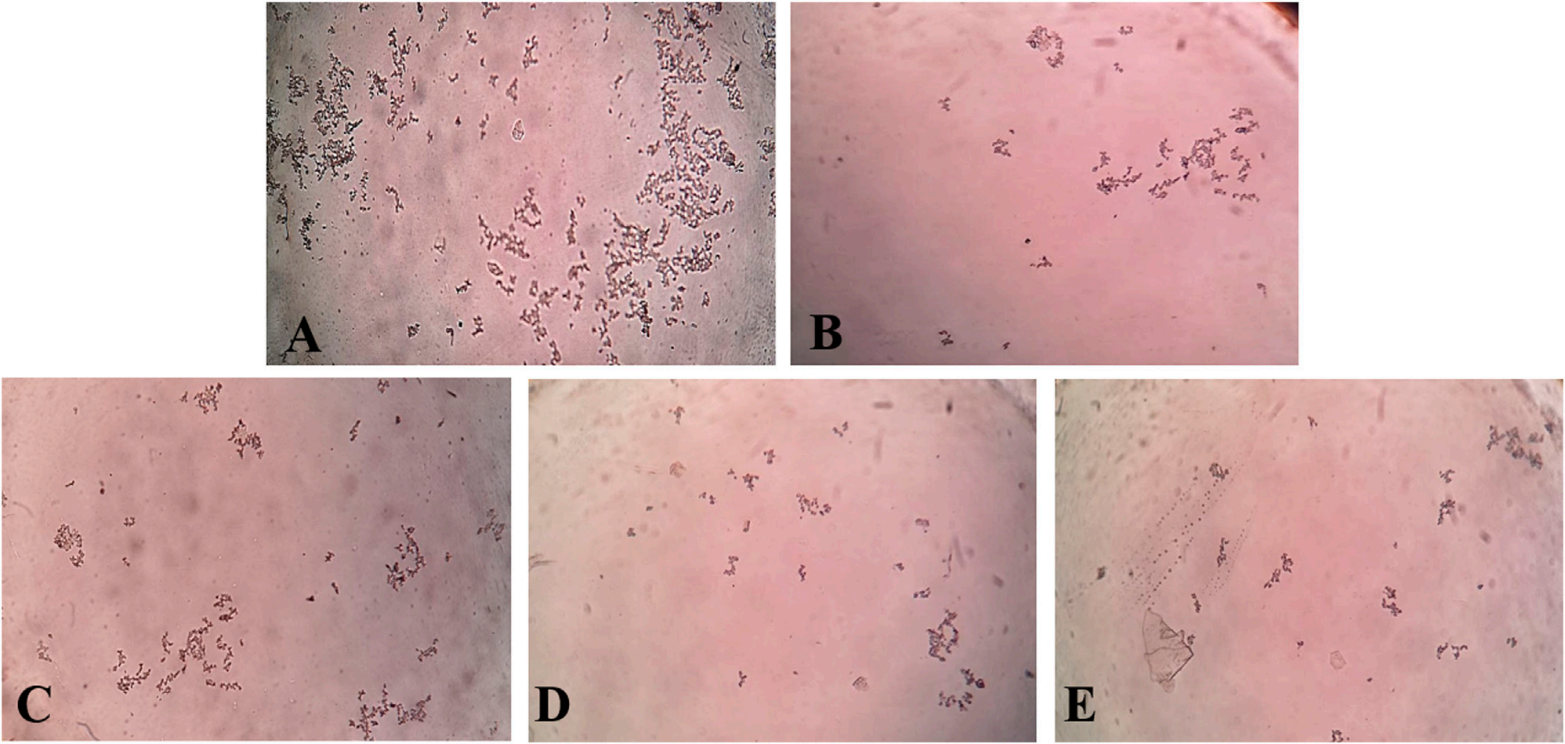
Photomicrograph of CaOx crystal density in different solutions. [**(A)** = Control, **(B)** = Cystone, **(C)** = AEEF (50 μg/mL), **(D)** = AEEF (100 μg/mL), **(E)** = AEEF (200 μg/mL)].

In the *in vitro* CaOx crystallization process, the optical density was raised to 10 min and then decreased to 30 min indicating the nucleation and aggregation processes, respectively, which is following the previous study (Hess et al., 2000). The change in the optical density of different solutions viz control, Cystone (1,000 μg/mL), AEEF (50, 100, and 200 μg/mL) were plotted at different time intervals (Figure 1A). Results revealed that AEEF (50, 100, and 200 μg/mL) and Cystone (1,000 μg/mL) inhibited the rate of nucleation (SN) while not significantly affecting the rate of aggregation (SA). The percent inhibition of the rate nucleation of CaOx by Cystone, AEEF (50 μg/mL), AEEF (100 μg/mL), and AEEF (200 μg/mL) were found to be 31.67, 21.98, 43.47, and 52.69 μg/mL, respectively (Figure 1B). The photographs of the different treatment crystal solutions were represented in Figure 2 which showed high-density CaOx crystals in control as compared to the standard drug (Cystone) as well as AEEF concentrations. This indicated that Cystone and AEEF inhibited the crystallization of CaOx crystals. The stones of CaOx mainly occur in two types CaOx monohydrate (COM) and CaOx dihydrate (COD). The commonly occurring and thermodynamically stable form is COM which is highly associated with renal tubular cells and may be accountable to form kidney stones. COM stones have a smooth surface and mainly occur in hyperoxaluria while COD stones jagged edges and are mainly related to hypercalciuria (Cloutier et al., 2015). Previous researchers stated that the test drug or extract could inhibit the crystallization by favoring the formation of COD crystals rather than COM (Fouda et al., 2006; Saha and Verma, 2013). The study needs the use of polarised light to differentiate between COM and COD crystals, which is a limitation of the present study and thus could not state whether the AEEF has more effect on COM or COD.

### 3.2 SiteMap analysis

For a better measurement of how much open the site is to the solvent, there were two properties were used. These were the two properties that measured how open the predicted site was to solvent. While the exposure determined the degrees of exposure of protein sites to solvent, the enclosure depicted the degrees of the enclosure by the protein sites. The contact score determined the amount of strength by which the predicted average site points interacted with the nearby receptor through van der Waals interactions. The phobic/philic scores depicted the predicted site’s relative hydrophobic and hydrophilic characteristics, respectively (Halgren 2007; Halgren 2009). On the other hand, the SiteScore and Dscore (Druggability Score) could be determined by the following equations:

$$SiteScore=0.0733\;sqrt\left(n\right)+0.6688\;e-0.20\;p$$

$$DScore=0.094\;sqrt\left(n\right)+0.60\;e-0.324\;p$$

Where *n* = no. of site points, *e* = Enclosure score, and *p* = hydrophilic score.

**FIGURE 3:**
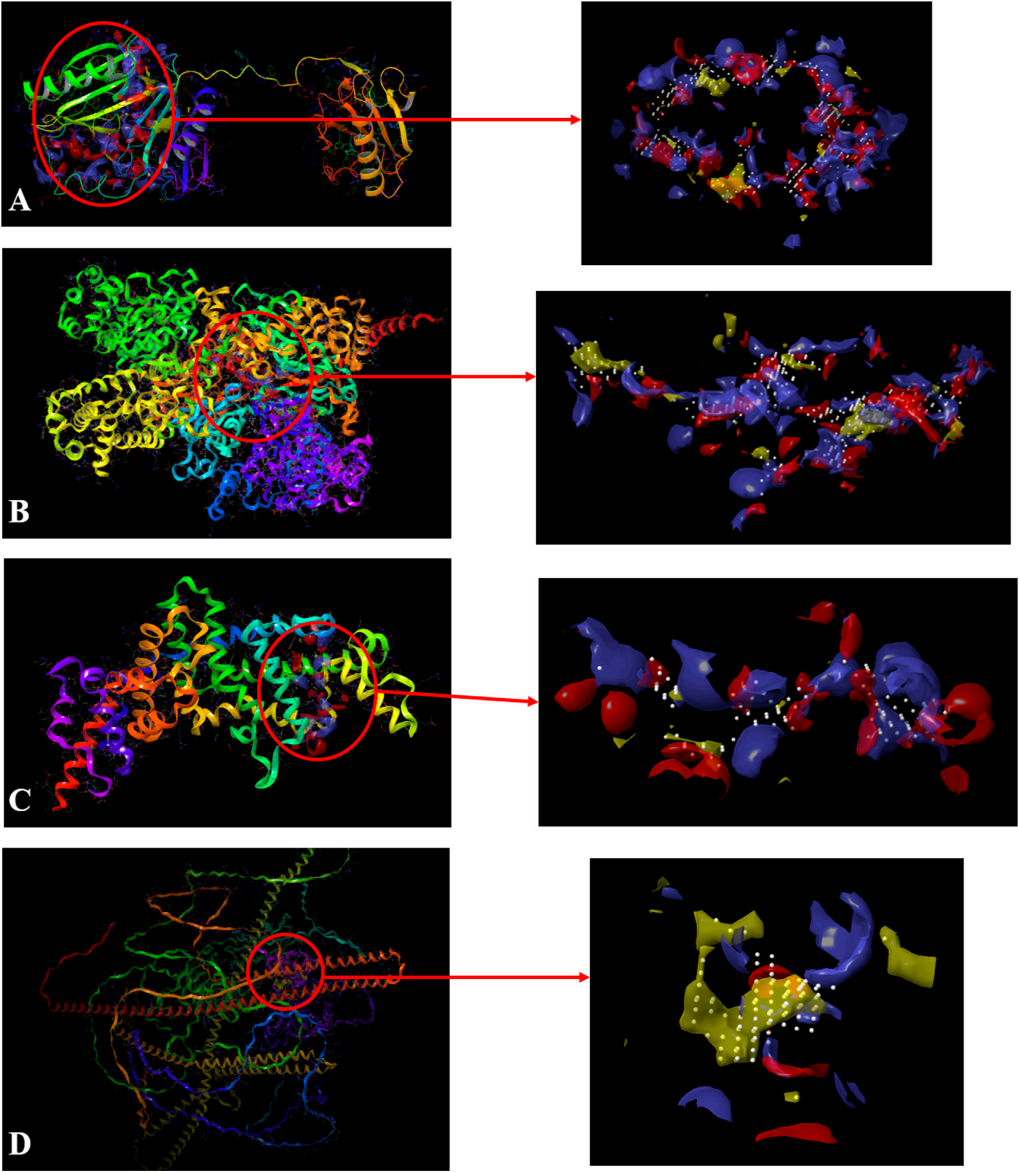
Sitemap-predicted site of Macrophage-capping protein (PDB ID: 1J72) **(A)**, UDP glucose: glycoprotein glucosyltransferase 2 (Gene: UGGT2) (AlphaFold) **(B)**, Ras GTPase-activating-like protein (PDB ID: 3FAY) **(C)**, RIMS-binding protein 3A (Gene: RIMBP3) (AlphaFold) **(D)**. (Yellow: Hydrophobic area, Blue: Hydrogen bond donor, Red: Hydrogen bond acceptor, White spheres: Site points).

In SiteMap Analysis, the properties were analyzed for the most favorable binding site of the Macrophage-capping protein (PDB ID: 1J72) interface using SiteMap (Schrödinger Release 2017_2). The site yielded a SiteScore of .99, a Dscore (druggability score) of .98, a volume of ∼1,405 Å^3^, and a total size of 406 Å^2^. The binding interface was found to have a phobic score of .375, a philic score of 1.13, and a balance score of .33 (Figure 3A). The contact score of this site was found to be .87, whereas the average score for a tight-binding site was 1.0. The exposure score was .6 and the enclosure score was found .68 whereas the average scores for a tight-binding site were .749, and .78 respectively. The don/acc score was 1.06. The hydrogen bond acceptor and donor regions refer to the degree that a well-structured ligand could interact with hydrogen bond donor and acceptor residues, respectively. The sitemap residues were Chain A: A132, A133, A134, A135, A136, A137, A139, A140, A142, A151, A152, A153, A154, A155, A156, A157, A158, A160, A163, A165, A168, A169, A170, A171, A173, A196, A197, A200, A201, A202, A203, A204, A205, A218, A221, A222, A223, A224, A230, A232, A233, A234, A235, A236, A237, A238, A239, A241, A242, A243, A244, A245, A246, A247, A248, A249, A251, A252, A254, A256, A257, A259, A261, A262, A263, A264, A265, A266, A267, A268, A269, A270, A271, A272, A275, A277, A280, A285, A286, A287, A288, A311, A314, A315, A318, A319, A321, and A343.

Similarly, the properties were analyzed for the most favorable binding site of the AlphaFold-predicted UDP glucose: glycoprotein glucosyltransferase 2 (Gene: UGGT2) (AlphaFold) using SiteMap (Schrödinger Release 2017_2) (see Methods). The site yielded a SiteScore of 1.06, a Dscore of 1.01, a volume of ∼992 Å^3^, and a total size of 375 Å^2^. The binding interface had a phobic score of .489 and a philic score of 1.224 and the Balance score of .4 (Figure 3B). The contact score of this site was 1.018, whereas the average score for a tight-binding site was 1.0. The exposure score was .49 and the enclosure score was .79 whereas the average scores for a tight-binding site were .749 and .78 respectively. The don/acc score was .792. The hydrogen bond acceptor and donor regions refer to the degree that a well-structured ligand could interact with hydrogen bond donor and acceptor residues, respectively. The sitemap residues are Chain A: A231, A232, A233, A235, A237, A384, A385, A388, A389, A390, A391, A392, A394, A395, A396, A398, A399, A402, A403, A935, A936, A937, A938, A939, A976, A977, A979, A980, A984, A1013, A1015, A1017, A1049, A1050, A1051, A1052, A1053, A1054, A1055, A1056, A1057, A1058, A1059, A1060, A1061, A1062, A1065, A1067, A1068, A1069, A1070, A1085, A1178, A1179, A1180, A1239, A1240, A1243, A1267, A1268, A1269, A1270, A1271, A1272, A1273, A1275, A1293, A1295, A1296, A1297, A1298, A1300, A1301, A1302, A1304, A1305, A1306, A1309, and A1313.

The properties were analyzed for the most favorable binding site of the Ras GTPase-activating-like protein (PDB ID: 3FAY) using SiteMap (Schrödinger Release 2017_2) (see Methods). The site yielded a SiteScore of .94, a Dscore of .86, a volume of ∼229 Å^3^, and a total size of 96 Å^2^. The binding interface had a phobic score of .21 and a philic score of 1.349 and the Balance score of .156 (Figure 3C). The contact score of this site was .899, whereas the average score for a tight-binding site was 1.0. The exposure score was .6 and the enclosure score was .64 whereas the average scores for a tight-binding site were .749, and .78 respectively. The don/acc score was .74. The hydrogen bond acceptor and donor regions refer to the degree that a well-structured ligand could interact with hydrogen bond donor and acceptor residues, respectively. The sitemap residues were Chain A: A1079, A1080, A1082, A1083, A1085, A1086, A1087, A1088, A1089, A1090, A1091, A1093, A1138, A1218, A1220, A1221, A1223, A1224, A1227, A1230, A1231, A1241, A1244, A1245, A1247, and A1248.

The properties were analyzed for the most favorable binding site of the RIMS-binding protein 3A (Gene: RIMBP3) (AlphaFold) using SiteMap (Schrödinger Release 2017_2) (see Methods). The site yielded a SiteScore of 1.03, a Dscore of 1.10, a volume of ∼148 Å^3^, and a total size of 86 Å^2^. The binding interface had a phobic score of 1.79 and a philic score of .54 and the Balance score of 3.279 (Figure 3D). The contact score of this site was .92, whereas the average score for a tight-binding site was 1.0. The exposure score was .56 and the enclosure score was .69 whereas the average scores for a tight-binding site were .749, and .78, respectively. The don/acc score was 2.34. The hydrogen bond acceptor and donor regions refer to the degree that a well-structured ligand could interact with hydrogen bond donor and acceptor residues, respectively. The sitemap residues were Chain A: A107, A110, A111, A113, A114, A115, A117, A188, A191, A192, A195, A196, A197, A198, A199, and A200.

### 3.3 Molecular docking analysis

The Glide XP docking scores and the amino acid residues responsible for the interactions were listed in Supplementary Tables S1, S2, respectively. Based on the docking scores the 3D and 2D interaction diagrams of the best five compounds were analyzed for the proposed activity. For the calculation of the binding free energy, the MMGBSA method was used.

**FIGURE 4:**
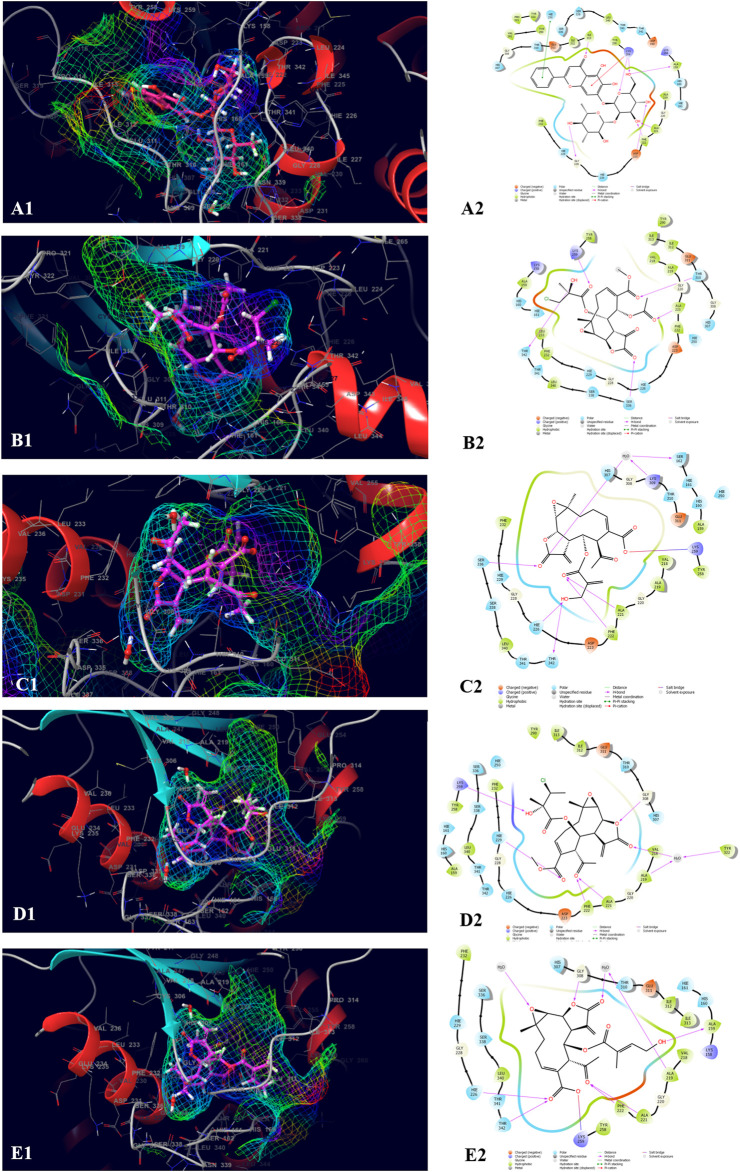
Molecular docking interactions of metabolites in the binding pocket of PDB ID: 3ELB. [3D and 2D representation of Baicalein 7-O-diglucoside **(A1,A2)**, 8-Deepoxyangeloyl-8-2-hydroxy-3-chloro- isobutyroyl-enhydrin **(B1,B2)**, 8-Desacylenhydrin-4-hydroxymethacrylate **(C1,C2)**, 8-Deepoxyangeloyl- 8-chloro- 2-hydroxy-2-methylbutyroylenhydrin **(D1,D2)** and 8-Desacyl enhydrin-4-hydroxytiglate **(E1,E2)** in the binding pocket of (PDB ID: 3ELB)].

The top five compounds which showed the best docking score and binding free energy against the PDB ID: 3ELB were found to be Baicalein 7-O-diglucoside (−14.073 kcal/mol, -35.896 kcal/mol), 8-Deepoxyangeloyl-8-2-hydroxy-3-chloro- isobutyroyl-enhydrin (−9.649 kcal/mol, −18.289 kcal/mol), 8-Desacylenhydrin-4-hydroxy methacrylate (−8.970 kcal/mol, -2.143 kcal/mol), 8-Deepoxyangeloyl- 8-chloro- 2-hydroxy-2-methylbutyroylenhydrin (−8.125 kcal/mol, 8.080 kcal/mol), and 8-Desacyl enhydrin-4-hydroxytiglate (−8.076 kcal/mol, -.763 kcal/mol). All the compounds had a docking score of above −8.000 kcal/mol which typically tells that they have the optimum interaction with the binding site. Results indicated that Ala 221, Phe 222, and Ser 336 were the most common amino acids which are responsible for the H-bond formation whereas Ala 159, Val 218, Ala 219, Ala 221, Tyr 258, and Leu 340 were the most common hydrophobic interactions forming amino acids present in the binding pocket. His 160, Hie 161, Hie 226, Hie 229, His 307, The 310, Ser 336, and The 342 were the amino acids accountable for the polar interactions. However, in some cases, the Lys 259 was able to form a weak water bridge (Figure 4).

**FIGURE 5:**
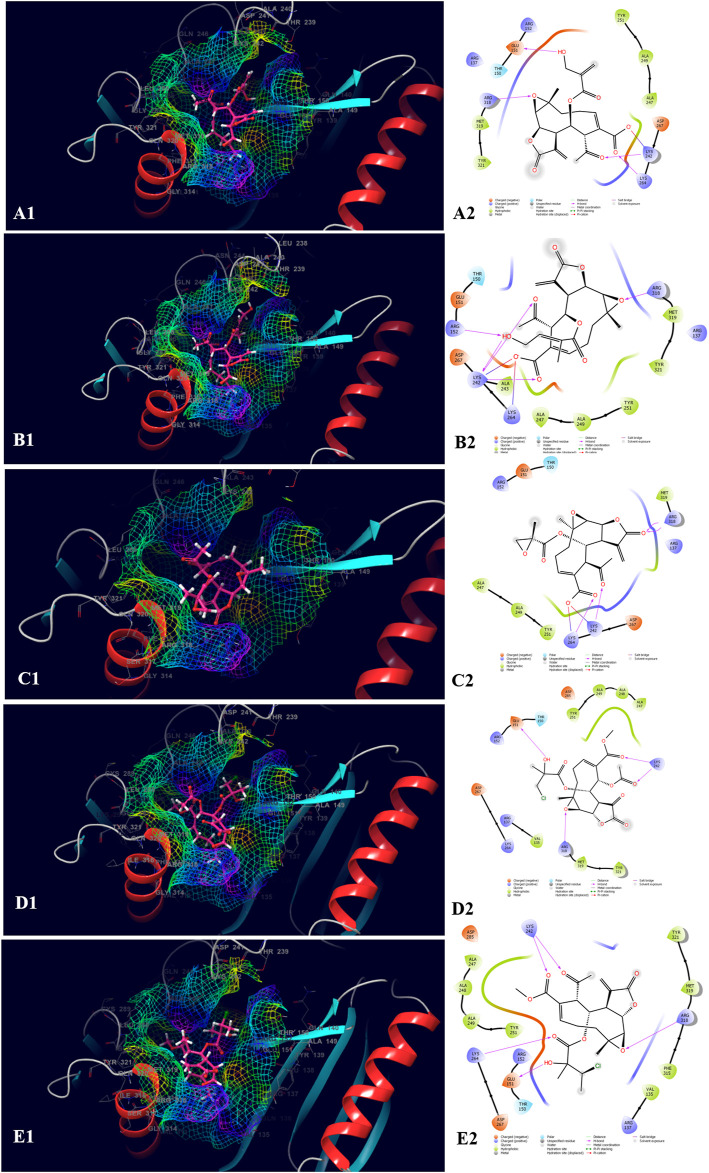
Molecular docking interactions of metabolites in the binding pocket of PDB ID: 1J72. [3D and 2D representation of 8-Desacylenhydrin-4-hydroxymethacrylate **(A1,A2)**, 8-Desacylenhydrin-4-hydroxytiglate **(B1,B2)**, 8-Desacylenhydrin-23-epoxyisobutyrate **(C1,C2)**, 8-Deepoxyangeloyl-8-2-hydroxy-3-chloro-isobutyroyl-enhydrin **(D1,D2)**, and 8-Deepoxyangeloyl-8-chloro-2-hydroxy2-methylbutyroylenhydrin **(E1,E2)** in the binding pocket of PDB ID: 1J72.].

The top five compounds which showed the best docking score and binding free energy against the PDB ID: 1J72 were 8-Desacylenhydrin-4-hydroxymethacrylate (−5.263 kcal/mol, −17.371 kcal/mol), 8-Desacylenhydrin-4-hydroxytiglate (-5.046 kcal/mol, -21.220 kcal/mol), 8-Desacylenhydrin-23-epoxyisobutyrate (−4.389 kcal/mol, −23.923 kcal/mol), 8-Deepoxyangeloyl-8-2-hydroxy-3-chloro-isobutyroyl-enhydrin (−4.317 kcal/mol, −18.572 kcal/mol), and 8-Deepoxyangeloyl-8-chloro-2-hydroxy2-methylbutyroylenhydrin (−3.833 kcal/mol, −21.318 kcal/mol). Lys 242 and Lys 264 were the amino acids responsible for the H-bond formation. Ala 247, Tyr 251, and Tyr 321 were the most common amino acids which are accountable for the hydrophobic interactions. The amino acid Thr 150 was the amino acid behind the polar interactions as well as water bridge formation in some cases (Figure 5).

**FIGURE 6:**
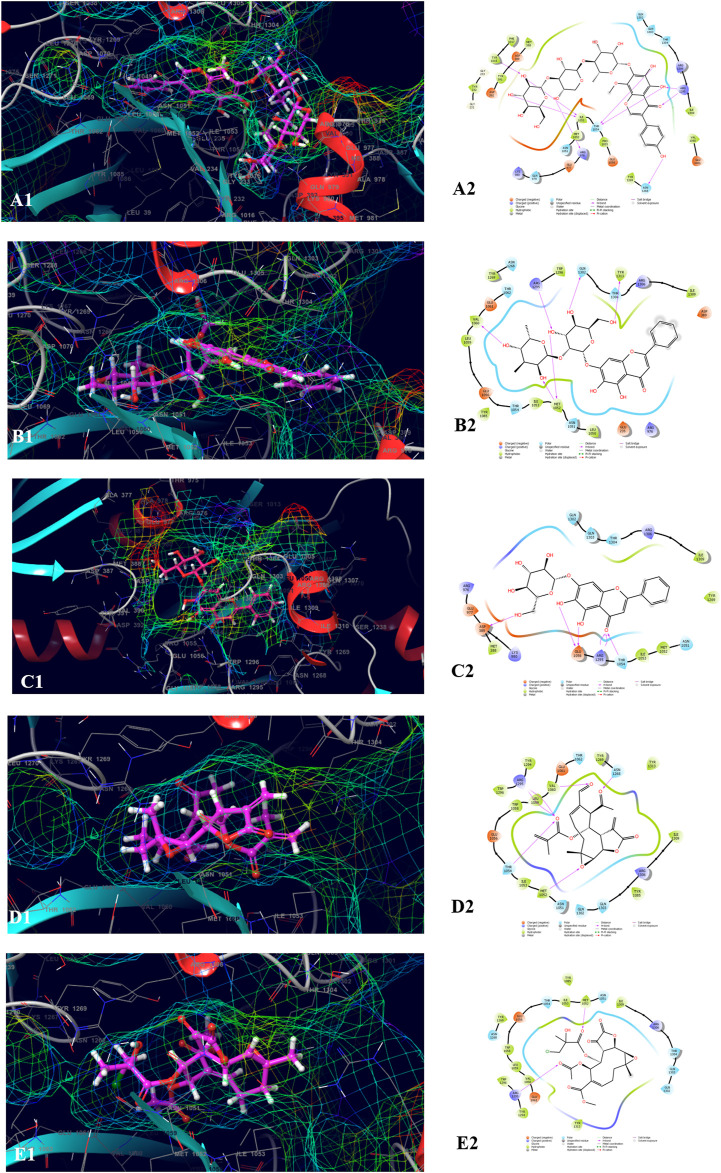
Molecular docking interactions of metabolites in the binding pocket of UDP glucose: glycoprotein glucosyltransferase 2 (Gene: UGGT2) (AlphaFold). [3D and 2D representation of 4′,5,6,7-Tetrahydroxy-8-methoxy isoflavone-7-O-beta-D-galactopyranosyl-(1→3)-O-beta-D-xylopyranosyl-(1→4)- O-alpha-L-rhamnopyranoside **(A1,A2)**, Baicalein-7-O-diglucoside **(B1,B2)**, Baicalein-7-O-glucoside **(C1,C2)**, 8-beta-Methacryloyloxy-9alpha-acetoxy-14oxoacanthospermolide **(D1,D2)**, and 8-Deepoxyangeloyl-8-2-hydroxy-3-chloro-isobutyroyl-enhydrin **(E1,E2)** in the binding pocket of UDP glucose:glycoprotein glucosyltransferase 2 (Gene: UGGT2) (AlphaFold)].

The top five compounds which showed the best docking score and binding free energy against UDP glucose: glycoprotein glucosyltransferase 2 (Gene: UGGT2) (AlphaFold) were 4′,5,6,7-Tetrahydroxy-8-methoxy isoflavone-7-O-beta-D-galactopyranosyl-(1→3)-O-beta-D-xylopyranosyl-(1→4)- O-alpha-L-rhamnopyranoside (−13.980 kcal/mol, −34.565 kcal/mol), Baicalein-7-O-diglucoside (−11.774 kcal/mol, −40.460 kcal/mol), Baicalein-7-O-glucoside (−8.950 kcal/mol, −18.883 kcal/mol), 8-beta-Methacryloyloxy-9alpha-acetoxy-14oxoacanthospermolide (-8.871 kcal/mol, −32.454 kcal/mol), and 8-Deepoxyangeloyl-8-2-hydroxy-3-chloro-isobutyroyl-enhydrin (−7.435 kcal/mol, −35.218 kcal/mol). All the above-mentioned compounds were found to have a considerable docking score. Lys 1,230 and Arg 1,295 were the amino acids responsible for the H-bond formation. Val 1,060, Tyr 1,269, and Ile 1,309 were the most common amino acids which are accountable for the hydrophobic interactions. Asn 1,268, Gln 1,302, and Thr 1,304 were responsible for the polar interactions (Figure 6).

**FIGURE 7:**
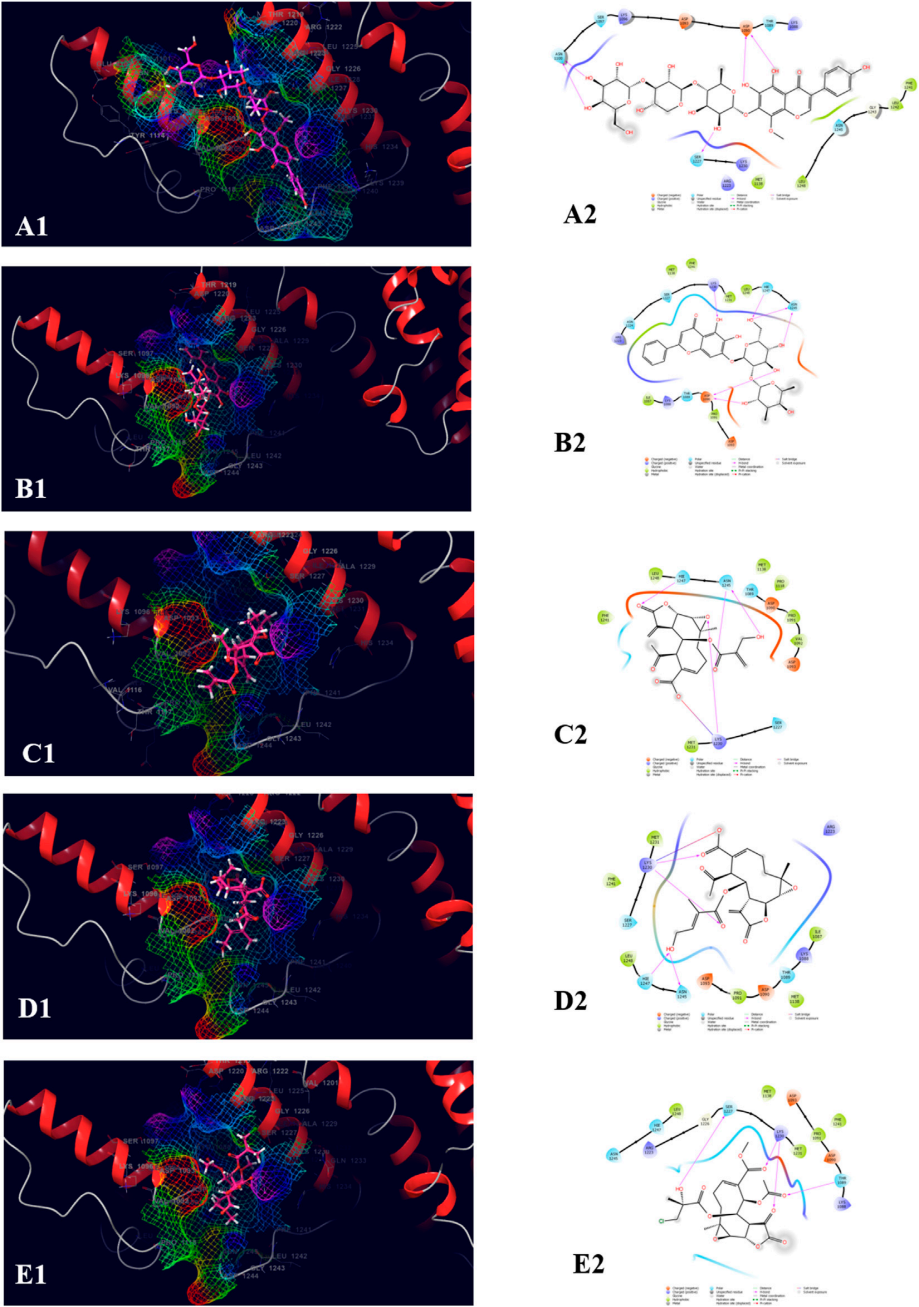
Molecular docking interactions of metabolites in the binding pocket of PDB ID: 3FAY. [3D and 2D representation of 4′,5,6,7-Tetrahydroxy-8-methoxyisoflavone-7-O-beta-D-galactopyranosyl-(1→3)-O-beta-D-xylopyranosyl-(1→4)- O-alpha-L-rhamnopyranoside **(A1,A2)**, Baicalein-7-O-diglucoside **(B1,B2)**, 8-Desacylenhydrin-4-hydroxymethacrylate **(C1,C2)**, 8-Desacylenhydrin-4-hydroxytiglate **(D1,D2)**, and 8-Deepoxyangeloyl-8-2-hydroxy-3-chloro-isobutyroyl-enhydrin **(E1,E2)** in the binding pocket of PDB ID: 3FAY].

The top five compound which showed the best docking score and binding free energy against PDB ID: 3FAY was 4′,5,6,7-Tetrahydroxy-8-methoxy isoflavone-7-O-beta-D-galactopyranosyl-(1→3)-O-beta-D-xylopyranosyl-(1→4)- O-alpha-L-rhamnopyranoside (−8.982 kcal/mol, −38.262 kcal/mol), Baicalein-7-O-diglucoside (−7.868 kcal/mol, −34.491 kcal/mol), 8-Desacylenhydrin-4-hydroxy methacrylate (−4.649 kcal/mol, −29.645 kcal/mol), 8-Desacylenhydrin-4-hydroxytiglate (−4.615 kcal/mol, −31.986 kcal/mol), and 8-Deepoxyangeloyl-8-2-hydroxy-3-chloro-isobutyroyl-enhydrin (−4.034 kcal/mol, −38.350 kcal/mol). The most common H-bond forming amino acids were Lys 1,230 and Hie 1,247 while Leu 1,248 and Phe 1,241 were the most common amino acids accountable for the hydrophobic interactions. Ser 1,227, Asn 1,245, and Hie 1,247 were found to be responsible for the polar interactions (Figure 7).

**FIGURE 8:**
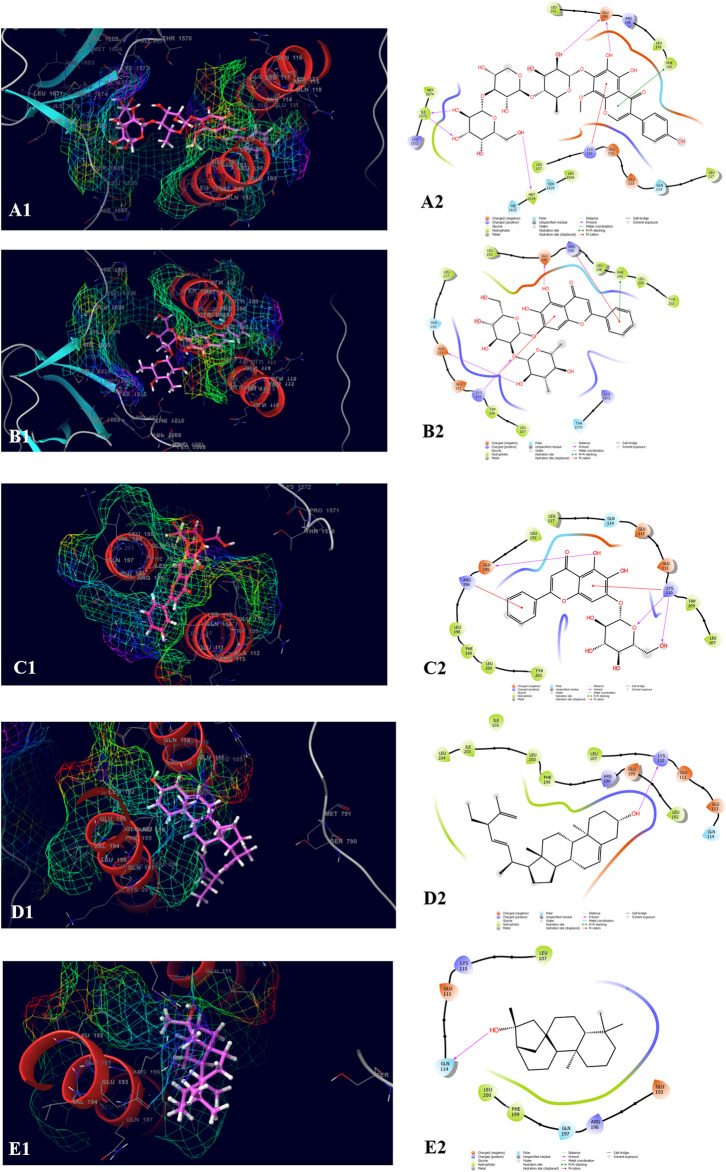
Molecular docking interactions of metabolites in the binding pocket of RIMS-binding protein 3A (Gene: RIMBP3) (AlphaFold). [3D and 2D representation of 4′,5,6,7-Tetrahydroxy-8-methoxyisoflavone-7-O-beta-D-galactopyranosyl-(1→3)-O-beta-D-xylopyranosyl-(1→4)- O-alpha-L-rhamnopyranoside **(A1,A2)**, Baicalein-7-O-diglucoside **(B1,B2)**, Baicalein-7-O-glucoside **(C1,C2)**, Stigmasta_5_22_25_trien_3beta_ol **(D1,D2)**, and Kauran_16_ol **(E1,E2)** in the binding pocket of RIMS-binding protein 3A (Gene: RIMBP3) (AlphaFold)].

The top five compounds which showed the best docking score and binding free energy against RIMS-binding protein 3A (Gene: RIMBP3) (AlphaFold) are 4′,5,6,7-Tetrahydroxy-8-methoxy isoflavone-7-O-beta-D-galactopyranosyl-(1→3)-O-beta-D-xylopyranosyl-(1→4)- O-alpha-L-rhamnopyranoside (−11.317 kcal/mol, −60.258 kcal/mol), Baicalein-7-O-diglucoside (−9.324 kcal/mol, −47.142 kcal/mol), Baicalein-7-O-glucoside (−7.376 kcal/mol, −39.360 kcal/mol), Stigmasta_5_22_25_trien_3beta_ol (−4.698 kcal/mol, −35.854 kcal/mol), and Kauran_16_ol (−4.562 kcal/mol, −33.901 kcal/mol). Lys 110 and Glu 195 were found the most common amino acids which are responsible for the H-bond formation. Leu 107 and Trp 109 were the most common hydrophobic interactions forming amino acids present in the binding pocket. Gln 114 was the amino acid accountable for the polar interactions. In some cases, the Lys 11 and Arg 196 were shown to form the water bridge (Figure 8).

### 3.4 Molecular dynamics

The molecular docking studies stipulated the importance of the interacting amino acids which were mentioned above. For the insight study of the interactions, there was a need for the molecular dynamics study of the best protein-ligand complexes which were performed for 50 ns of the simulation time. For a better understanding of the solidity and visualization of the period of the interacting amino acids in the dynamics condition, the protein-ligand RMSD as well as protein-ligand contacts were explored. The validation of the stability of the receptor-ligand complex was done by performing molecular dynamics simulations using Desmond of Schrödinger 2017-2 for 50 ns.

**FIGURE 9:**
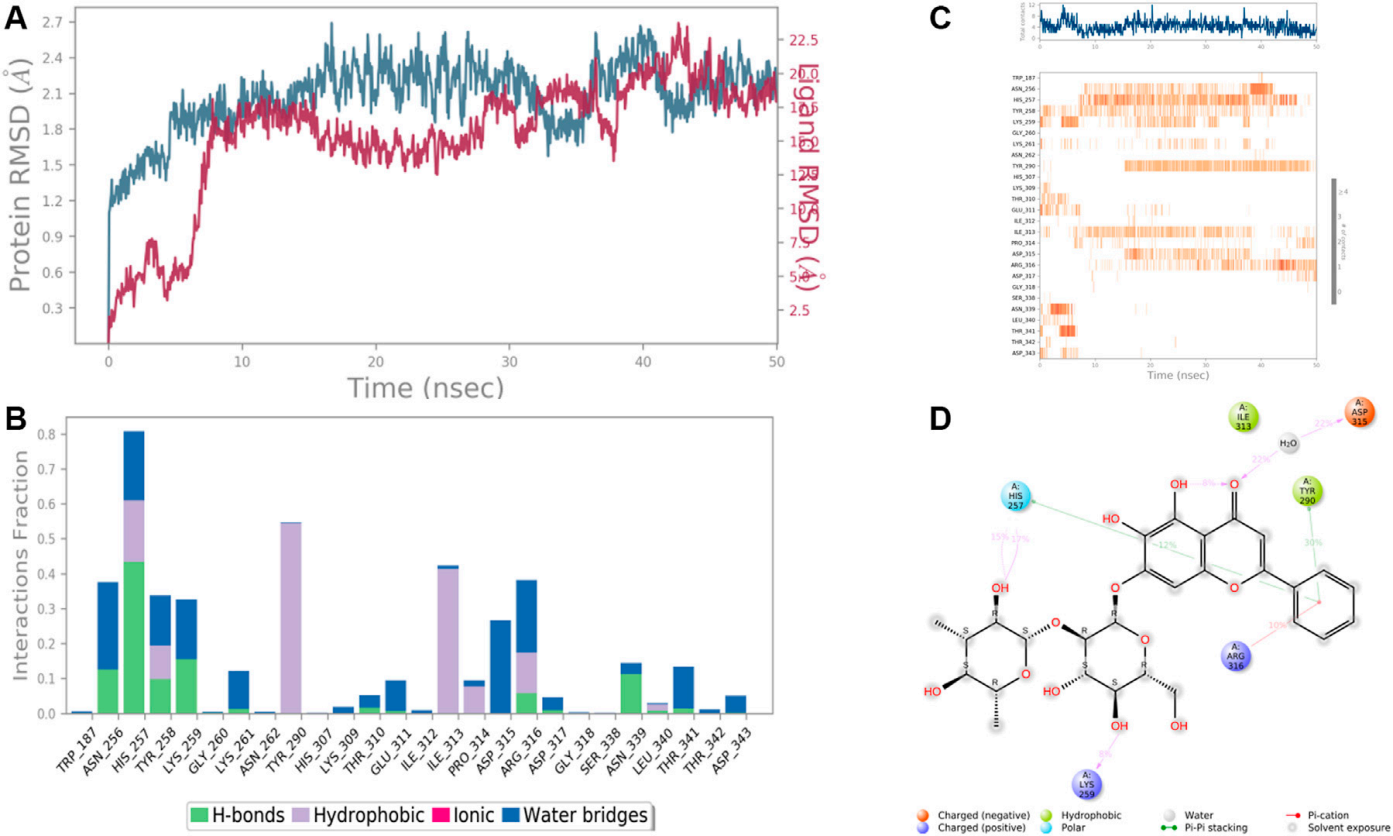
RMSD plot of the protein-ligand complex (PDB ID: 3ELB) **(A)**, Protein-ligand contacts **(B)**, stacked bar plot of the fraction of time of the interactions **(C)**, and Ligand- Protein contacts **(D)** of analogue Baicalein 7-O-diglucoside for 50 ns of simulation time.

Results revealed that His 257, Lys 259, Tyr 290, Ile 313, and Asp 315, etc. were found the important amino acids for binding of the ligands inside the binding pocket of the Ethanolamine-phosphate cytidylyltransferase. The protein and ligand RMSD (Å) was found to be < 1.5 Å which validated the stability of the analog Baicalein 7-O-diglucoside at the active site of the protein (PDB ID: 3ELB) (Figure 9). During the start of the simulation (0–10 ns), there was a bit of divergence (<1.2 Å). Moreover to demonstrate the protein-ligand stability the interactions were simulated for 50 ns. The amino acids Tyr 290 and Ile 313 were involved in the hydrophobic interactions for a maximum simulation time of .4 and .5 fractions of time, respectively. Moreover the important amino acid Asn 256 was involved in H-bonding, hydrophobic and water bridge formation for .4, .2, and .2 fractions of the total simulation time.

**FIGURE 10:**
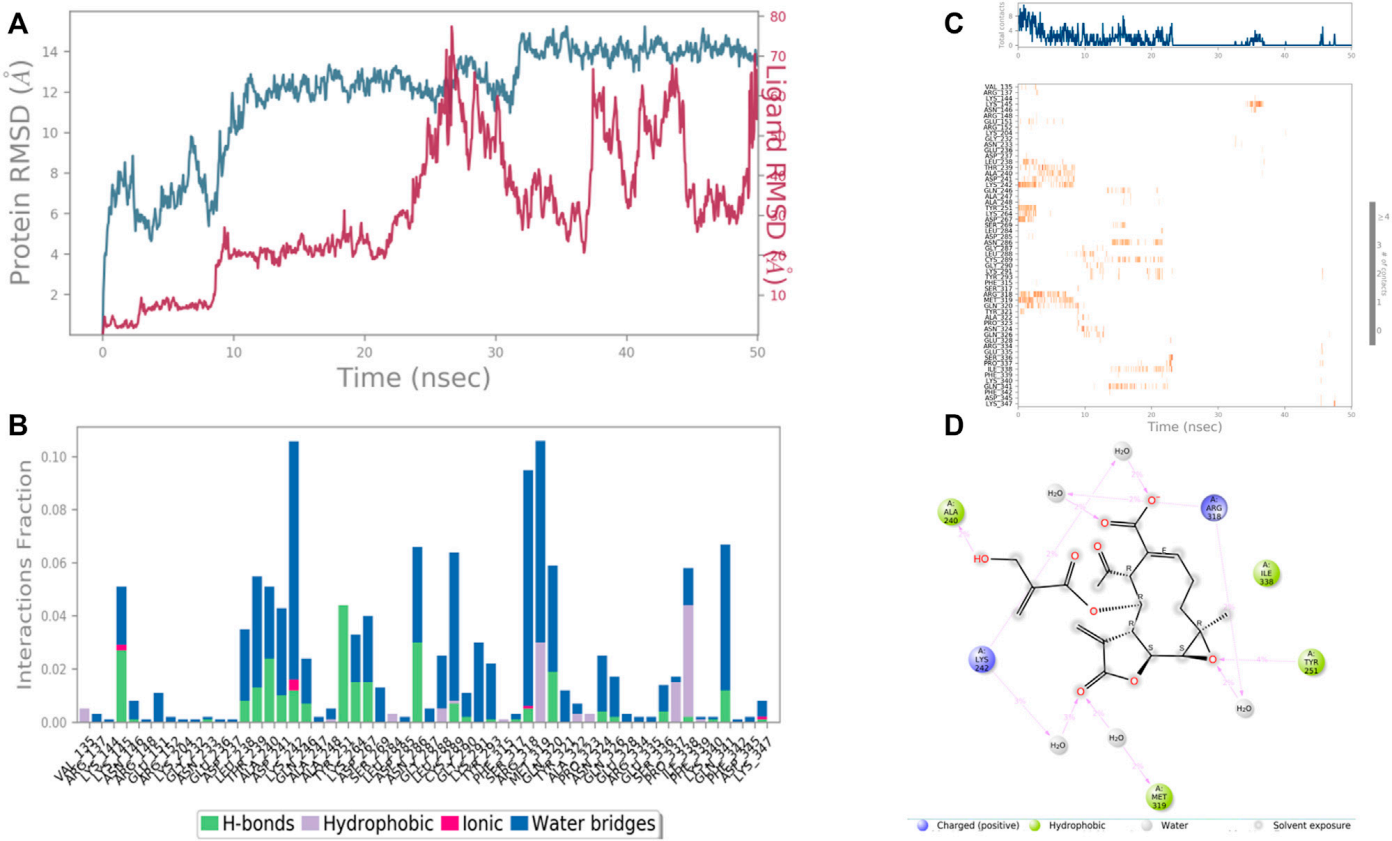
RMSD plot of the protein-ligand complex (PDB ID: 1J72) **(A)** Protein-ligand contacts **(B)**, stacked bar plot of the fraction of time of the interactions **(C)** and Ligand- Protein contacts **(D)** of analogue 8-Desacyl enhydrin-[4-hydroxymethacrylate] for 50 ns of simulation time.

In the case of Macrophage-capping protein (PDB ID: 1J72) the 8-Desacyl enhydrin [4-hydroxy methacrylate] was found to be interacting with the Ala 240, Lys 242, Gln 242, Tyr 251, Arg 318, Met 319, Ile 338 in the binding site of the particular protein (PDB ID: 1J72). During the start of the simulation (0–25 ns), there was a bit of divergence (<8 Å). Moreover to demonstrate the protein-ligand stability the interactions were simulated for 50 ns (Figure 10). The amino acid Gln 242 was involved in the hydrophobic interactions as well as water bridge formation for a maximum simulation time of .02 and .08 fraction of time, respectively. Most of the amino acids were responsible for weak water bridge formation. Tyr 251 was responsible for the hydrophobic interactions with a simulation time of .04 fraction of time.

**FIGURE 11:**
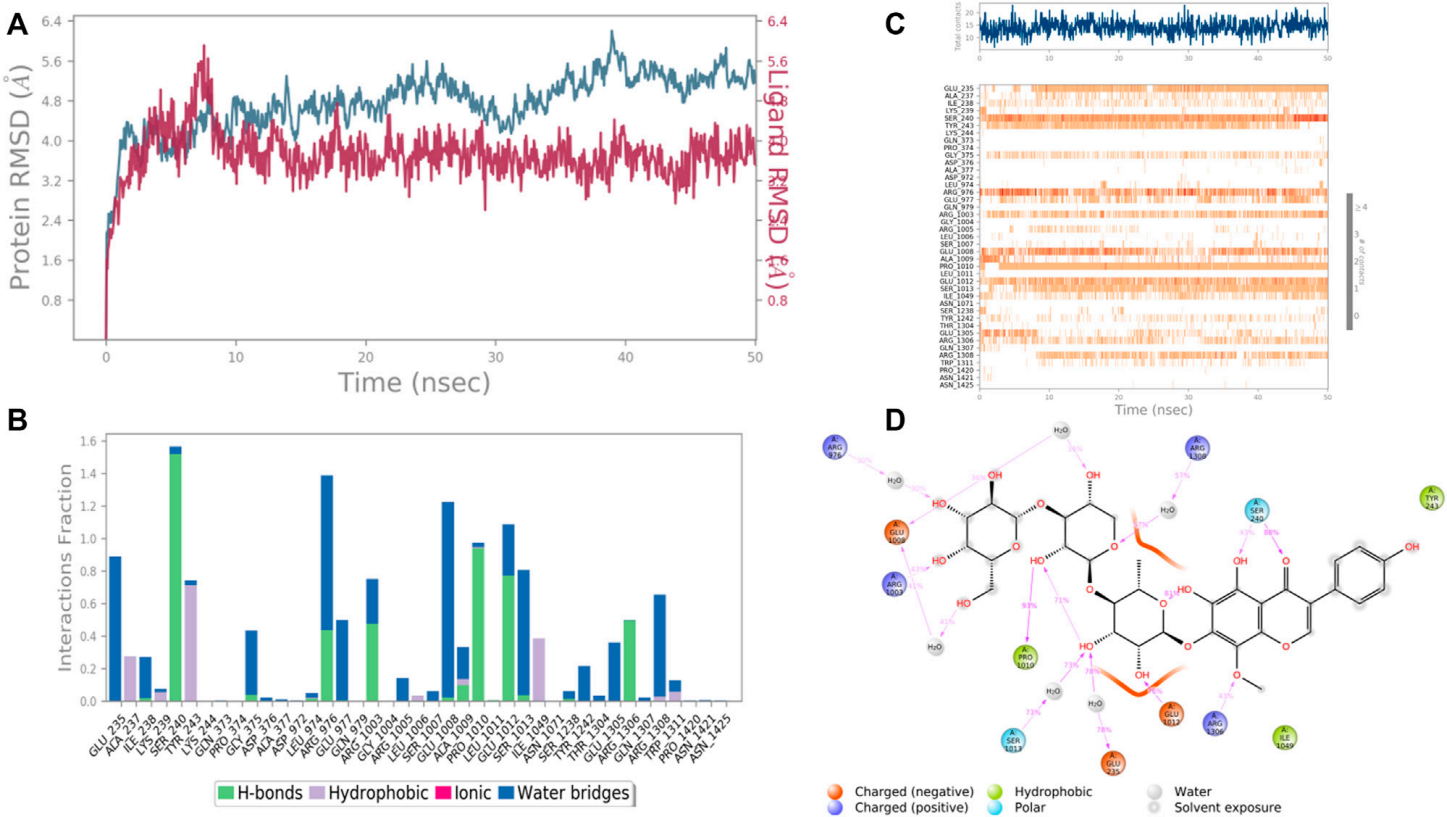
RMSD plot of the protein–ligand complex UDP glucose:glycoprotein glucosyltransferase 2 (Gene: UGGT2) (AlphaFold) **(A)**, Protein-ligand contacts **(B)**, stacked bar plot of the fraction of time of the interactions **(C)** and Ligand- Protein contacts **(D)** of analogue 4′,5,6,7-Tetrahydroxy-8-methoxy isoflavone-7-O-beta-D-galactopyranosyl-(1→3)-O-beta-D-xylopyranosyl-(1→4)- O-alpha-L-rhamnopyranoside for 50 ns of simulation time.

In case of UDP glucose: glycoprotein glucosyltransferase 2 (Gene: UGGT2) (AlphaFold), the interacting amino acid residues with 4′,5,6,7-Tetrahydroxy-8-methoxy isoflavone-7-O-beta-D-galactopyranosyl-(1→3)-O-beta-D-xylopyranosyl-(1→4)- O-alpha-L-rhamnopyranoside were Glu 235, Ser 240, Arg 1,308, Pro 1,010, and Glu 1,012 etc. in the binding site. From the starting point till the completion of the whole simulation, the protein-ligand complex was found to be quite stabilized. Ser 240 and Glu 1,008 were the H-bonding and water bridges forming residues for a maximum simulation time of 1.5 and 1.2 fractions of time, respectively. Also, another water bridge-forming residue Glu 235 was found to be quite stable for .8 fractions of the time of the simulation study. Most of the amino acids were responsible for weaker water bridges forming interaction. Additionally, Pro 1,010 was the both H-bond and water-bridge forming residue for 1.0 fraction of the time of the simulation study. Tyr 243 and Ile 1,049 were the hydrophobic residues for .7 and .4 fractions of time (Figure 11).

**FIGURE 12:**
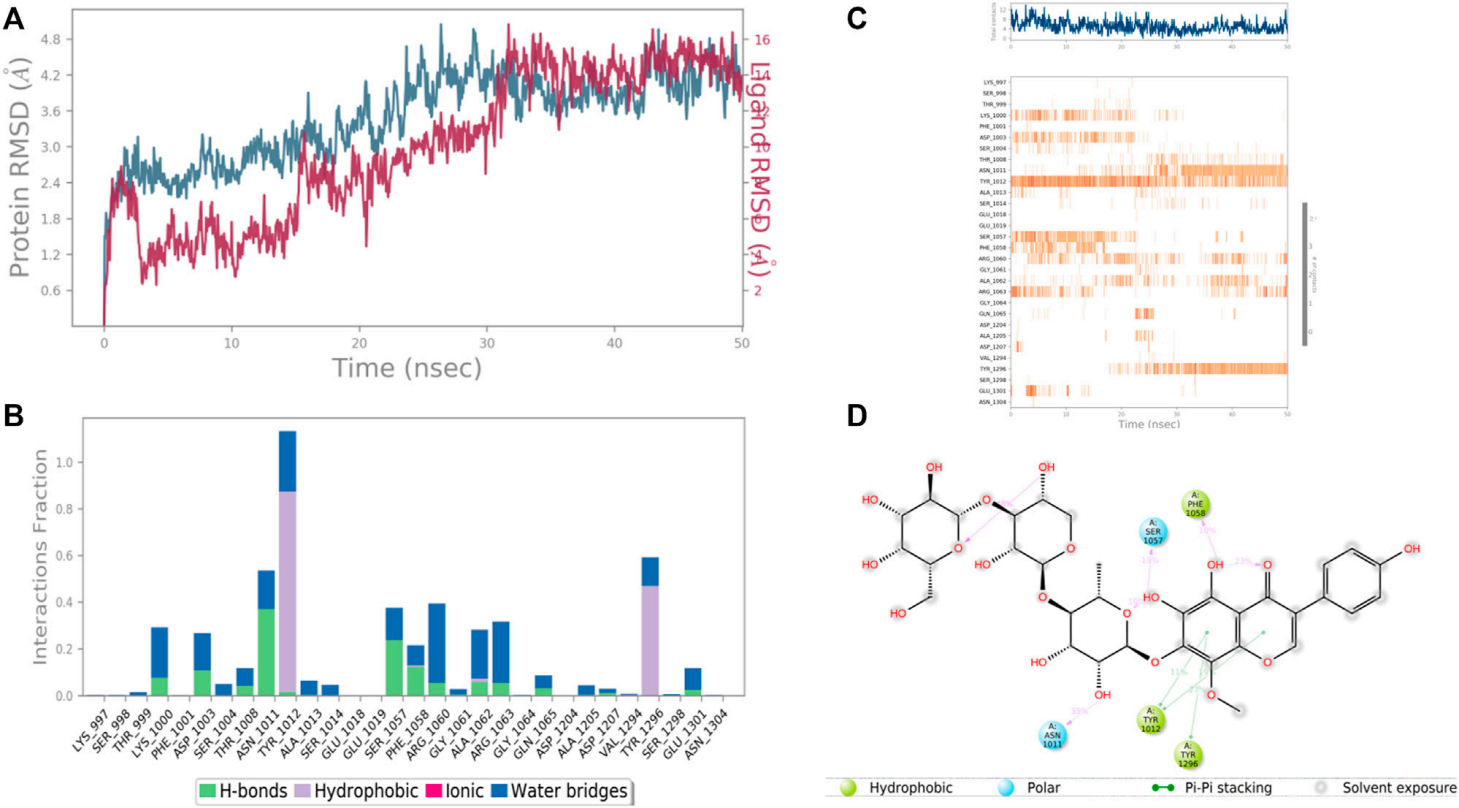
RMSD plot of the protein–ligand complex (PDB ID: 3FAY) **(A)**, Protein-ligand contacts **(B)**, stacked bar plot of the fraction of time of the interactions **(C)** and Ligand- Protein contacts **(D)** of analogue 4′,5,6,7-Tetrahydroxy-8-methoxyisoflavone-7-O-beta-D-galactopyranosyl-(1→3)-O-beta-D-xylopyranosyl-(1→4)- O-alpha-L-rhamnopyranoside for 50 ns of simulation time.

In the case of Ras GTPase-activating-like protein, the interacting amino acid residues with 4′,5,6,7-Tetrahydroxy-8-methoxy isoflavone-7-O-beta-D-galactopyranosyl-(1→3)-O-beta-D-xylopyranosyl-(1→4)- O-alpha-L-rhamnopyranoside were Ser 1,057, Phe 1,058, Asn 1,011, Tyr 1,012, and Tyr 1,296, etc. in the binding site. From the starting point till the completion of the whole simulation, the protein-ligand complex was found to be quite stabilized. Tyr 1,012 and Tyr 1,296 were the hydrophobic residues for .9 and .5 fraction of time of the simulation study, respectively. These two residues were also hydrophobic interaction forming residues for 1.0 and .6 fraction of time, respectively. Most of the amino acids were responsible for weaker water bridges forming interaction. Among the H-bonding residues, Asn 1,011 was the most stabilized one for a .4 fraction of time (Figure 12).

**FIGURE 13:**
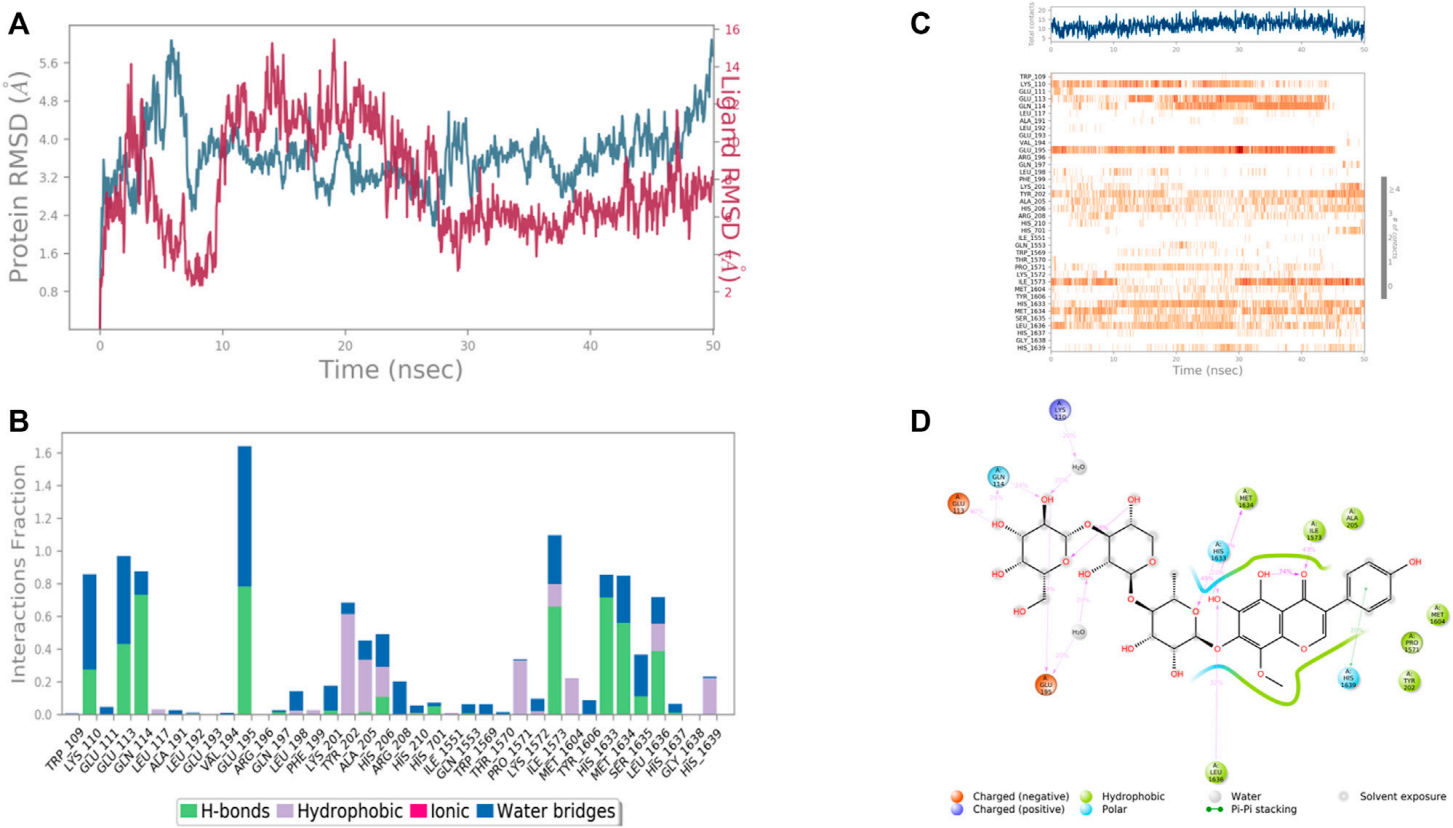
RMSD plot of the protein–ligand complex [RIMS-binding protein 3A (Gene: RIMBP3) (AlphaFold)) **(A)**, Protein-ligand contacts **(B)**, stacked bar plot of the fraction of time of the interactions **(C)** and Ligand- Protein contacts **(D)** of analogue 4′,5,6,7-Tetrahydroxy-8-methoxyisoflavone-7-O-beta-D-galactopyranosyl-(1→3)-O-beta-D-xylopyranosyl-(1→4)- O-alpha-L-rhamnopyranoside for 50 ns of simulation time.

In case of RIMS-binding protein 3A (Gene: RIMBP3) (AlphaFold), the interacting amino acid residues with 4′,5,6,7-Tetrahydroxy-8-methoxy isoflavone-7-O-beta-D-galactopyranosyl-(1→3)-O-beta-D-xylopyranosyl-(1→4)- O-alpha-L-rhamnopyranoside were Glu 113, Gln 114, Glu 195, His 1,633, Met 1,634, and Leu 1,636 etc. in the binding site. From the starting point till the completion of the whole simulation, the protein-ligand complex was found to be quite stabilized. Glu 114, Glu 195, Ile 1,573, Tyr 1,606 and His 1,633 were the H bonding residues for .75, .75, .7, .8, and .7 fractions of time of the simulation study, respectively. Tyr 202 was the hydrophobic interaction forming residues for .6 fraction of time. Among the water bridges forming residues Lys 110 and Glu 113 were the most stabilized ones for .5 fraction of time (Figure 13).

### 3.5 In silico ADMET profile

The best two molecules which had satisfactory docking scores against all of the four proteins mentioned above were the pyranose moiety named Baicalein-7-O-diglucoside, 4′,5,6,7-Tetrahydroxy-8-methoxy isoflavone-7-O-beta-D- galactopyranosyl-(1→3)-O-beta-D-xylopyranosyl-(1→4)- O-alpha-L-rhamnopyranoside. The *in silico* ADME/T properties were predicted using the QikProp program of Schrödinger 2017-2, and were tabulated in Supplementary Table S3. All of these properties were found to be in the acceptable range. Both of the molecules were not found to be CNS-active. Both of the molecules showed acceptable hydrophobic solvent-accessible surface areas of 297.946 and 360.561 A^3, respectively. All-in-all, both molecules can be processed for further studies due to their drug-likeness and considerable pharmacokinetic property with very less Lipinski Rule of 5 and Jorgensen Rule of 3 violations.

### 3.6 Pharmacophore modeling

**FIGURE 14:**
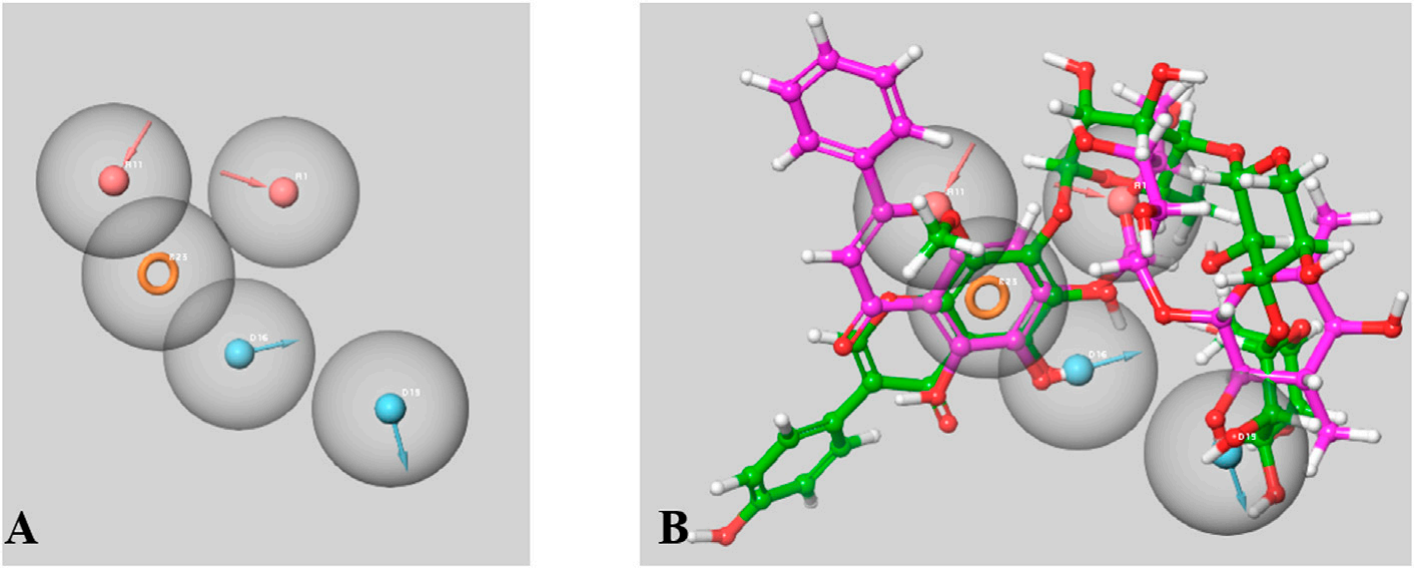
A Five-point Pharmacophore model (AADDR_3) generated by PHASE **(A)**. The model illustrates acceptor feature (red sphere), donar feature (blue sphere) and aromatic rings (orange ring) features. Both active phtoconstituents Baicalein-7-O-diglucoside (represented in magenta) and 4′,5,6,7-Tetrahydroxy-8-methoxyisoflavone-7-O-beta-D- galactopyranosyl-(1→3)-O-beta-D-xylopyranosyl-(1→4)- O-alpha-L-rhamnopyranoside (represented in green) overlapped on the generated model AADDR_3 **(B)**.

There were 15 pharmacophoric hypotheses developed as depicted in Supplementary Table S4, and post-development, all of them were ranked based on their PhaseHypoScore, Survival Score, Site Score (range .0–1.0), Vector Score (range −1.0 to 1.0), Volume Score (range .0–1.0), BEDROC Score, and Fitness Score (range −1.0–3.0). The Fitness Score was referred to as the linear combination of Volume Score, Vector Score, and Site Score, where the Fitness Score of 3 was considered the exact match. The top-ranked hypothesis was obtained as a five-point pharmacophore (AADDR_3) consisting of two H-bond acceptors (A), two H-bond donors (D), and one aromatic ring (R) (Figure 14), where all these scores were found to be in the acceptable range. The Survival Score, Site Score, Vector Score, Volume Score, BEDROC Score, and PhaseHypoScore were 3.453, .547, .798, .363, 1.000, and 1.207, respectively for this top-ranked hypothesis. Here, the Fitness Scores of 4′,5,6,7-Tetrahydroxy-8-methoxyisoflavone-7-O-beta-D-galactopyranosyl-(1→3)-O-beta-D-xylopyranosyl-(1→4)- O-alpha-L-rhamnopyranoside and Baicalein 7-O-diglucoside were found to be 1.708 and 3.000, respectively.

## 4 Conclusion

In conclusion, the results of the present investigations suggest that the aqueous extract of *E. fluctuans* Lour. has an *in vitro* anti-crystallization effect on CaOx crystals. Further, *in silico* studies suggests the modulatory effects of two polyphenolic coumarin ring-containing metabolites Baicalein-7-O-diglucoside and 4′,5,6,7-Tetrahydroxy-8-methoxy isoflavone-7-O-beta-D-galactopyranosyl-(1→3)-O-beta-D-xylopyranosyl-(1→4)-O-alpha-L-rhamnopyranoside on the four renal stone matrix-associated protein (Human CTP: Phosphoethanolamine Cytidylyltransferase (PDB ID: 3ELB), UDP glucose: glycoprotein glucosyltransferase 2 (Gene: UGGT2) (AlphaFold) and RIMS-binding protein 3A (Gene: RIMBP3) (AlphaFold), and Ras GTPase activating-like protein (PDB: 3FAY) which may inhibit the crystallization process. Further bioactivity-guided fractionations and *in-vivo* studies along with molecular studies like western blot/ELISA/Proteomics are needed to confirm and strengthen the anti-urolithiatic activity of *E. fluctuans* as well as to elucidate the possible mechanism of action involved therein.

## Data Availability

The datasets presented in this study can be found in online repositories. The names of the repository/repositories and accession number(s) can be found in the article/Supplementary Material.

## Glossary

Acc: Accepter
ADME/T: Absorption, distribution, metabolism, excretion, and toxicity
AEEF: Aqueous extract of *Enhydra fluctuans*
BSI: Botanical Survey of India
CaOx: Calcium oxalate
CNS: Central nervous system
COD: Calcium oxalate dihydrate
COM: Calcium oxalate monohydrate
Conc: Concentration
Don: Donner
*E. fluctuans*: *Enhydra fluctuans*
ELISA: Enzyme-linked immunosorbent assay
g: Gram
h: Hour
H-bond: Hydrogen bond
K: Kelvin
ml: Millilitre
mM: Millimole
min: Minute
MMGBSA: Molecular mechanics-generalized Born surface area
MD: Molecular dynamics
NaCl: Sodium chloride
NPT: Standard temperature and pressure
no: Number
ns: Nano second
nm: Nanometre
pH: Potential of Hydrogen
PDB: Protein data bank
rpm: Revolutions per minute
SA: Rate of aggregation
SASA: Solvent accessible surface area
S.D.: Standard deviation
Sec: Second
SN: Rate of nucleation
VdW: Van der Waals
Viz: Videlicet
2D: Two dimensional
3D: Three dimensional
°**C**: Degree Celsius
%: Percentage
µg: Microgram
Å: Angstrom
