# Supplementary material for: Inhibitory activity of Enhydra fluctuans Lour. on calcium oxalate crystallisation through in silico and in vitro studies
Source: Front Pharmacol. 2023 Jan 20;13:982419. doi: 10.3389/fphar.2022.982419 (PMC9894874; doi:10.3389/fphar.2022.982419)
Supplement: Supplementary file 1 [file DataSheet1.docx]

**Supplementary materials**

**Table S1. Molecular docking results of the phytoconstituents present in the *E. fluctuans* based on their docking scores**

| **Compound Name** | **Ethanolamine-**  **phosphate cytidylyltransferase (PDB ID: 3ELB)**  **GlideScore (Kcal/mol)** | **Macrophage-capping**  **Protein**  **(PDB ID: 1J72)**  **GlideScore (Kcal/mol)** | **UDP-**  **glucose:glycoprotein glucosyltransferase 2**  **(UDP glucose:glycoprotein glucosyltransferase 2 (Gene: UGGT2) (AlphaFold))**  **GlideScore (Kcal/mol)** | **Ras GTPase-activating-like protein**  **(PDB ID: 3FAY)**  **GlideScore (Kcal/mol)** | **RIMS-binding protein 3A**  **(RIMS-binding protein 3A (Gene: RIMBP3) (AlphaFold))**  **GlideScore (Kcal/mol)** |
| --- | --- | --- | --- | --- | --- |
| Enhydrin | -4.743 | -2.175 | -3.091 | -3.793 | -3.202 |
| Fluctuadin | -4.916 | -2.128 | -2.709 | -2.035 | -2.529 |
| Fluctuanin | -4.590 | -2.767 | -3.665 | -2.142 | -2.727 |
| beta-Carotene | - | - | 0.913 | 0.344 | -2.637 |
| Baicalein 7-O-glucoside | - | - | **-8.950** | - | **-7.376** |
| alpha-Pinene | -3.419 | - | -2.814 | -1.354 | -3.257 |
| 1-Octen-3-ol | -3.416 | - | -2.439 | -1.289 | -2.760 |
| Myrcene | -2.147 | - | -1.902 | -1.402 | -2.562 |
| Limonene | -3.331 | - | -1.790 | -1.556 | -3.235 |
| (E)-beta-Ocimene | -3.051 | - | -0.478 | -1.423 | -2.898 |
| Linalool | -4.226 | - | -3.242 | -1.675 | -2.995 |
| Camphor | -3.662 | - | -1.346 | -1.763 | -2.875 |
| cis-1,2-Dihydroperillaldehyde | -5.021 | - | -4.676 | -1.918 | -3.252 |
| Perillaldehyde | -4.675 | - | -3.265 | -2.116 | -3.491 |
| (E)-Caryophyllene | -3.514 |  | -3.557 | -0.985 | -3.385 |
| alpha-Humulene | -3.719 | -2.511 | -4.198 | -0.834 | -3.247 |
| Longiverbenone | -4.623 | - | -4.126 | -1.114 | -2.991 |
| Stigmasterol | - | - | -5.094 | -1.803 | -3.255 |
| Stigmasta-5,22,25-trien-3beta-ol | - | - | -5.129 | -0.537 | **-4.698** |
| Myricyl alcohol | - | - | -4.272 | -3.423 | -3.065 |
| (-)-Kauran-16-ol | -5.099 | - | -5.607 | -2.388 | **-4.562** |
| -)-Kaur-16-en-19-oic acid | -4.082 | - | -4.091 | -2.616 | -3.041 |
| Gibberellin A9 | -4.007 | - | -6.222 | -2.490 | -3.106 |
| Gibberellin A13 | -7.036 | - | -5.022 | -2.725 | -3.065 |
| 8-Deepoxyangeloyl-8-[2-hydroxy-3-chloro-isobutyroyl]- enhydrin | **-9.649** | **-4.317** | **-7.435** | **-4.034** | -4.203 |
| 4-Hydroxyfarnesyl acetate | -1.897 | - | -5.009 | -1.388 | -4.055 |
| 8-Desacyl enhydrintiglate | -7.767 | -3.011 | -6.110 | -3.484 | -3.476 |
| 8-Desacyl enhydrin-[4-hydroxymethacrylate] | **-8.970** | **-5.263** | -6.996 | **-4.649** | -3.874 |
| 8-Desacyl enhydrin-[4-hydroxytiglate] | **-8.076** | **-5.046** | -5.590 | **-4.615** | -3.601 |
| 8-Desacyl enhydrin-[2,3-epoxyisobutyrate] | -7.581 | **-4.388** | -6.087 | -3.303 | -4.379 |
| 8-beta-Methacryloyloxy-9alpha-acetoxy-14oxoacanthospermolide | -6.788 | -3.436 | **-8.871** | -3.303 | -3.146 |
| Baicalein 7-O-diglucoside | **-14.073** | - | **-11.774** | **-7.868** | **-9.316** |
| 4',5,6,7-Tetrahydroxy-8-methoxyisoflavone-7-O-beta-D-galactopyranosyl-(1→3)-O-beta-D-xylopyranosyl-(1→4)- O-alpha-L-rhamnopyranoside | -7.520 | - | **-13.980** | **-8.982** | **-11.317** |
| 8-Deepoxyangeloyl-8-[chloro-2-hydroxy-2-methylbutyroyl]-enhydrin | **-8.125** | **-3.833** | -6.475 | -3.918 | -3.877 |
| Myricic alcohol | - | - | - | - | - |

**Table S2: Molecular docking results of the compounds present in the *E. fluctuans* based on their interactions**

| **Protein name** | **Compound Name** | **H-bond forming residues** | **Hydrophobic interaction forming residues** | **Polar interaction forming residues** | **Water bridge forming residues** | **Pi-pi stacking/Pi-cation interaction forming residues** |
| --- | --- | --- | --- | --- | --- | --- |
| Ethanolamine-phosphate cytidylyltransferase (PDB ID:3ELB) | Baicalein-7-O-diglucoside | Ala 159, Ala 221, Phe 222, Asp 223, Gly 228, Lys 259, Ile 313 | Ala 219, Gly 220, Ala 221, Phe 222, Phe 232, Tyr 258, Tyr 290, Val 292, Pro 321, Tyr 322, Ile 312, Ile 313, | Hie 160, Hie 161, Hie 226, Hie 229, Hie 250, His 307, The 310 Ser 336, Ser 338, Thr 341, Thr 342, | Lys 259 | Hie 250, Lys 259 |
|  | 8-Deepoxyangeloyl-8- 2-hydroxy-3-chloroisobutyroyl-enhydrin | Ala 221, Lys 259, Thr 310, Ser 336 | Ala 159, Val 218, Ala 219, Gly 220, Ala 221, Phe 222, Phe 232, Leu 233, Tyr 258, Tyr 290, Ile 312, Ile 313, Leu 340 | Hie 160, Hie 161, Hie 226, Hie 229, Hie 250, His 307, Thr 310, Hie 336, Hie 338 Thr 341, The 342 | - | - |
|  | 8-Desacylenhydrin-4- hydroxymethacrylate | Ala 221, Phe 222, Hie 226, Lys 309, Ser 336, The 342 | Ala 159, Val 218, Ala 219, Ala 221, Phe 222, Phe 232, Tyr 258, Leu 340 | His 160, Hie 161, Ser 162, Hie 226, Hie 229, Hie 250, His 307, Thr 310, Ser 336, Ser 338, Thr 341, Thr 342 | Lys 259 | - |
|  | 8-Deepoxyangeloyl-8- chloro-2-hydroxy2- methylbutyroylenhydrin | Ala 219, Ala 221, Phe 222, Hie 229, Lys 259, Gly 308 | Ala 159, Val 218, Ala 219, Ala 221, Phe 222, Phe 232, Tyr 258, Tyr 290, Ile 312, Ile 313, Tyr 322, leu 340 | His 160, Hie 161, Hie 226, Hie 229, Hie 250, His 307, Thr 310, Ser 336, Ser 338, Thr 341, Thr 342 | - | - |
|  | 8-Desacyl enhydrin-4- hydroxytiglate | Ala 159, Ala 219, Ala 221, Phe 222, Hie 226, lys 259, Gly 308, Thr 342 | Ala 159, Val 218, Ala 219, Ala 221, Phe 222, Phe 232, Tyr 258, ILe 312, Ile 313, Leu 340 | His 160, Hie 161, Hie 226, Hie 229, His 307, Thr 310, Ser 336, Ser 338, Thr 341, Thr 342 | Lys 259 | - |
| Macrophage Capping Protein (Cap G) (PDB ID: 1J72) | 8-Desacylenhydrin-4- hydroxymethacrylate | Glu 151, Lys 242, Lys 264, Arg 318 | Ala 247, Ala 249, Tyr 251, Met 319, Tyr 321 | Thr 150 | Lys 242 | - |
|  | 8-Desacylenhydrin-4- hydroxytiglate | Arg 152, Lys 242, Lys 264 | Ala 243, Ala 247, Ala 249, Tyr 251, , Met 319, Tyr 321 | Thr 150 | Lys 242, Lys 264 | - |
|  | 8-Desacylenhydrin-23- epoxyisobutyrate | Lys 242, Lys 264, Arg 318 | Ala 247, Ala 249, Ala 251, Met 319 | Thr 150 | Lys 262, Lys 264 | - |
|  | 8-Deepoxyangeloyl-8- 2-hydroxy-3-chloroisobutyroyl-enhydrin | Glu 151, Lys 242, Arg 318 | Val 135, Ala 247, Ala 248, Tyr 251,Tyr 321, Met 319 | Thr 150 | - | - |
|  | 8-Deepoxyangeloyl-8- chloro-2-hydroxy2- methylbutyroylenhydrin | Glu 151, Lys 242 Lys 264, Arg 318 | Val 135, Ala 247, Ala 248, Ala 249, Tyr 251, Phe 315, Met 319, Tyr 321 | Thr 150 | - | - |
| UDP glucose:glycoprotein glucosyltransferase 2 (Gene: UGGT2) (AlphaFold) | 4',5,6,7-Tetrahydroxy-8-methoxyisoflavone-7-O-beta-D-galactopyranosyl-(1→3)-O-beta-D-xylopyranosyl-(1→4)- O-alpha-L-rhamnopyranoside | Arg 976, Tyr 1015, Ile 1053, Arg 1295 | Tyr 232, Met 388, Tyr 391, Tyr 1015, Phe 1017, Met 1052, Ile 1053, val 1060, Ile 1309 | Gln 979, Asn 1051, Thr 1054, Asn 1268, Gln 1302, Gln 1303, Thr 1304 | - | - |
|  | Baicalein-7-O-diglucosi | Met 1052, Val 1060, Arg 1295, Gln 1302, Tyr 1313, | Leu 1050, Met 1052, Ile 1053, Leu 1059, VAl 1060, Tyr 1085 | Asn 1051, Thr 1054, Thr 1062, Asn 1268, Gln 1302, Thr 1304 | - | - |
|  | Baicalein-7-O-glucoside | Asp 389, Thr 1054, Glu 1056, Arg 1295 | Met 388, Met 1052, Ile 1053, Tyr 1269, Ile 1309 | Asn 1051, Thr 1054, Gln 1302, Gln 1303, Thr 1304 | - | - |
|  | 8-beta-Methacryloyloxy-9alpha-acetoxy-14oxoacanthospermolide | Met 1052, Thr 1054, Val 1060, Asn 1268, Arg 1295 | Met 1052, Ile 1053, Trp 1058, Leu 1059, Val 1060, Tyr 1085, Tyr 1269, Trp 1296, Tyr 1294, Tyr 1309, Tyr 1313 | Asn 1051 Thr 1054, Thr 1062, Asn 1268, Gln 1302, Gln 1303 | - | - |
|  | 8-Deepoxyangeloyl-8-2- hydroxy-3-chloroisobutyroyl-enhydrin | Met 1052, Arg 1295 | Met 1052, Ile 1053, Trp 1058, Leu 1059, Val 1060, Tyr 1085, Tyr 1269, Tyr 1294, Trp 1296, Ile 1309, Tyr 1313 | Asn 1051, Thr 1054, Gln 1302, Gln 1303, Thr 1304, asn 1368 | - | - |
|  | 8-Desacylenhydrin-4- hydroxymethacrylate | Thr 1094, Tyr 1294, arg 1295 | Met 1052, Ile 1053, Leu 1059, Val 1060, Tyr 1085, Tyr 1269, Tyr 1294, Trp 1296, Tyr 1313 | Asn 1051, Thr 1054, Thr 1062, Asn 1268, Gln 1302 | Arg 1295 | - |
| Ras GTPase activating-like protein (PDB ID: 3FAY) | 4',5,6,7-Tetrahydroxy-8-methoxyisoflavone-7-O-beta-D-galactopyranosyl-(1→3)-O-beta-D-xylopyranosyl-(1→4)- O-alpha-L-rhamnopyranoside | Asp 1000, Asn 1100 | Met 1138, Phe 1241, Leu 1242, Leu 1248 | Thr 1089, Ser 1097, Asn 1100, Ser 1227, Asn 1245 | - | - |
|  | Baicalein-7-O-diglucosid | Asp 1090, Lys 1230, Asn 1245, Hie 1247, | Ile 1087, Pro 1091, Met 1138, Met 1231, Phe 1241, Leu 1248 | Thr 1089, Asn 1224, Ser 1227, Asn 1245, Hie 1247 | - | - |
|  | 8-Desacylenhydrin4- hydroxymethacrylate | Lys 1230, Asn 1245, Hie 1247 | Pro 1091, Val 1092, Pro 1118, Met 1138, Met 1231, Phe 1241, Leu 1248 | Thr 1089, Ser 1227, Asn 1245, Hie 1247 | Lys 1230 | - |
|  | 8-Desacylenhydrin4-hydroxytiglate | Lys 1230, Asn 1245, Hie 1247 | Ile 1087, Pro 1091, Met 1138, Met 1231, Phe 1241, Leu 1248 | Thr 1089, Ser 1227, Asn 1245, Hie 1247 | Lys 1230 | - |
|  | 8-Deepoxyangeloyl-8-2-hydroxy-3- chloro-isobutyroylenhydrin | Thr 1089, Lys 1230, Ser 1227 | Pro 1091, Met 1138, Met 1231, Phe 1241, Leu 1248 | Thr 1089, Ser 1227, Hie 1247, Asn 1245 | - | - |
| RIMS-binding protein 3A (Gene: RIMBP3) (AlphaFold) | 4',5,6,7-Tetrahydroxy-8-methoxyisoflavone-7-O-beta-D-galactopyranosyl-(1→3)-O-beta-D-xylopyranosyl-(1→4)- O-alpha-L-rhamnopyranoside | Lys 110, Glu 195, Ile 1573, Met 1634 | Leu 107, Leu 117, Leu 192, Leu 198, Leu 199, Ile 1573, Met 1574, Met 1636 | Gln 114, Ser 1635, Hie 1633 | - | Lys 110, Phe 199 |
|  | Baicalein-7-O-diglucoside | Lys 110, Glu 113, Glu 195, | Leu 107, Trp 109, Leu 117, Leu 192, Leu 198, Phe 199, Leu 200, Tyr 202 | Gln 114, Thr 1570 | - | Lys 110, Arg 196, Phe 199 |
|  | Baicalein-7-O-glucoside | Lys 110, Glu 195 | Leu 107, Trp 109, Leu 117, Leu 192, Leu 198, Phe 199, Leu 200, Tyr 202 | Gln 114 | - | Lys 110, Arg 196 |
|  | Stigmasta_5_22_25_trien_3beta_ol | Lys 110 | Ile 103, Leu 107, Leu 192, Phe 199, Leu 200, Ieu 203, Leu 204 | Gln 114 | - | - |
|  | Kauran_16_ol | Gln 114 | Leu 107, Phe 199, Phe 200 | Gln 114, Gln 197 | - | - |
|  | 8-Desacylenhydrin-23- epoxyisobutyrate | Gln 118, Arg 125, Gln 189, Arg 196 | Leu 121, Leu 192, Trp 792 | Gln 114, Gln 118, His 122, Gln 189 | Arg 196 | - |

**Table S3. Physico-chemical properties and ADME/T profile**

| **Compound** | **Hydrophobic SASA** | **CI QP log S** | **QP log BB** | **QP polrz** | **QP log S** | **volume** |
| --- | --- | --- | --- | --- | --- | --- |
| Baicalein-7-O-diglucoside | 297.946 | -4.872 | -3.216 | 52.684 | -3.78 | 1600.175 |
| 4',5,6,7-Tetrahydroxy-8-methoxyisoflavone-7-O-beta-D-galactopyranosyl-(1→3)-O-beta-D-xylopyranosyl-(1→4)- O-alpha-L-rhamnopyranoside | 360.561 | -4.266 | -6.26 | 62.07 | -2.532 | 1976.024 |

SASA - solvent-accessible surface area, CI QP log S - Predicted conformation independent solubility, QP log BB - Predicted blood-brain barrier permeability, QP polrz - Predicted Polarizability, QP log S - Predicted aqueous solubility

**Table S4. Various pharmacophore hypotheses generated by PHASE.**

| Hypothesis | Survival Score | Site Score | Vector Score | Volume Score | BEDROC Score | PhaseHypoScore | Fitness Score |
| --- | --- | --- | --- | --- | --- | --- | --- |
| **AADDR_3** | 3.453 | 0.547 | 0.798 | 0.363 | 1.000 | 1.207 | **3.000^*^, 1.708^**^** |
| ADDR_1 | 3.444 | 0.709 | 0.856 | 0.354 | 1.000 | 1.206 | 3.000^*^, 1.921^**^ |
| AADR_1 | 3.426 | 0.752 | 0.926 | 0.358 | 1.000 | 1.205 | 2.036^*^, 3.000^**^ |
| AAADR_1 | 3.400 | 0.559 | 0.851 | 0.362 | 1.000 | 1.204 | 1.773*, 3.000** |
| AAADR_3 | 3.378 | 0.529 | 0.800 | 0.367 | 1.000 | 1.202 | 3.000^*^, 1.697^**^ |
| ADDRR_2 | 3.376 | 0.264 | 0.831 | 0.358 | 1.000 | 1.202 | 1.453^*^, 3.000^**^ |
| AADR_2 | 3.308 | 0.694 | 0.851 | 0.349 | 1.000 | 1.198 | 1.895^*^, 3.000^**^ |
| AADDR_2 | 3.462 | 0.547 | 0.798 | 0.363 | 0.990 | 1.197 | 1.708^*^, 3000^**^ |
| ADR_1 | 3.296 | 0.973 | 0.918 | 0.236 | 1.000 | 1.197 | 2.128^*^, 3.000^**^ |
| AAADR_2 | 3.379 | 0.529 | 0.800 | 0.367 | 0.990 | 1.193 | 1.697^*^, 3.000^**^ |
| AADR_3 | 3.289 | 0.782 | 0.827 | 0.346 | 0.990 | 1.187 | 1.956^*^, 3.000^**^ |
| AAAR_2 | 3.383 | 0.706 | 0.932 | 0.361 | 0.980 | 1.183 | 2.000^*^, 3.000^**^ |
| AAAR_1 | 3.421 | 0.883 | 0.853 | 0.354 | 0.971 | 1.176 | 2.091^*^, 3.000^**^ |
| AADDR_1 | 3.470 | 0.484 | 0.813 | 0.352 | 0.952 | 1.161 | 3.000^*^, 1.649^**^ |
| AAR_3 | 3.181 | 0.820 | 0.849 | 0.324 | 0.962 | 1.152 | 1.995^*^, 3.000^**^ |

^*^Baicalein-7-O-diglucoside ^**^4',5,6,7-Tetrahydroxy-8-methoxyisoflavone-7-O-beta-D- galactopyranosyl-(1→3)-O-beta-D-xylopyranosyl-(1→4)- O-alpha-L-rhamnopyranoside
